# Non-invasive detection of orthotopic human lung tumors by microRNA expression profiling of mouse exhaled breath condensates and exhaled extracellular vesicles

**DOI:** 10.20517/evcna.2023.77

**Published:** 2024-03-29

**Authors:** Megan I. Mitchell, Iddo Z. Ben-Dov, Christina Liu, Tao Wang, Rachel B. Hazan, Thomas L. Bauer, Johannes Zakrzewski, Kathryn Donnelly, Kar Chow, Junfeng Ma, Olivier Loudig

**Affiliations:** ^1^Center for Discovery and Innovation, Hackensack Meridian Health, Nutley, NJ 07110, USA.; ^2^Laboratory of Medical Transcriptomics, Hadassah-Hebrew University Medical Center, Jerusalem 91120, Israel.; ^3^Department of Epidemiology and Population Health, The Albert Einstein College of Medicine, Montefiore Medical Center, Bronx, NY 10461, USA.; ^4^Department of Pathology, The Albert Einstein College of Medicine, Montefiore Medical Center, Bronx, NY 10461, USA.; ^5^Jersey Shore University Medical Center, Hackensack Meridian Health, Neptune City, NJ 07753, USA.; ^6^Hackensack University Medical Center, Hackensack Meridian Health, Hackensack, NJ 07601, USA.; ^7^Department of Oncology, Lombardi Comprehensive Cancer Center, Georgetown University Medical Center, Washington, DC 20007, USA.

**Keywords:** Extracellular vesicles, exhaled breath condensate, orthotopic lung tumor-bearing animal model, miRNAs

## Abstract

**Aim:**

The lung is the second most frequent site of metastatic dissemination. Early detection is key to improving survival. Given that the lung interfaces with the external environment, the collection of exhaled breath condensate (EBC) provides the opportunity to obtain biological material including exhaled miRNAs that originate from the lung.

**Methods:**

In this proof-of-principal study, we used the highly metastatic MDA-MB-231 subline 3475 breast cancer cell line (LM-3475) to establish an orthotopic lung tumor-bearing mouse model and investigate non-invasive detection of lung tumors by analysis of exhaled miRNAs. We initially conducted miRNA NGS and qPCR validation analyses on condensates collected from unrestrained animals and identified significant miRNA expression differences between the condensates of lung tumor-bearing and control mice. To focus our purification of EBC and evaluate the origin of these differentially expressed miRNAs, we developed a system to collect EBC directly from the nose and mouth of our mice.

**Results:**

Using nanoparticle distribution analyses, TEM, and ONi super-resolution nanoimaging, we determined that human tumor EVs could be increasingly detected in mouse EBC during the progression of secondary lung tumors. Using our customizable EV-CATCHER assay, we purified human tumor EVs from mouse EBC and demonstrated that the bulk of differentially expressed exhaled miRNAs originate from lung tumors, which could be detected by qPCR within 1 to 2 weeks after tail vein injection of the metastatic cells.

**Conclusion:**

This study is the first of its kind and demonstrates that lung tumor EVs are exhaled in mice and provide non-invasive biomarkers for detection of lung tumors.

## INTRODUCTION

Lung cancer is the second leading cause of cancer incidence (2.1 million cases per year) and mortality (1.8 million deaths per year) globally^[1,2]^. However, the lung is also the second most frequent site of metastatic growth for extra-thoracic malignancies^[3]^. It is recognized that because of its role in blood circulation, the lung offers optimal conditions for the development of secondary cancers that arise from primary colorectal (~25.8%), head and neck (~19.4%), urologic (i.e., bladder, kidney, and testicular; ~14.7%), breast (~10.5%), melanoma (~6.5%), gynecological, blood, and other cancers (~6.1%)^[4]^. Currently, it is estimated that ~5%-10% of patients with malignant cancer will at some point develop pulmonary metastatic lesions that are either synchronous (i.e., found at the time of primary cancer diagnosis) or metachronous (i.e., found as a recurrent lesion or after primary cancer diagnosis)^[5,6]^. The 5-year survival rate for patients who develop secondary lung metastases is very low; for example, it is estimated at ~21% for patients initially diagnosed with primary breast cancer and < 10% for those initially diagnosed with primary colorectal cancer^[7,8]^. Clinically, the early detection of micro-metastases developing within the lung tissue is extremely difficult as early invading cells are disseminated and their physical detection by chest x-rays or computed tomography (CT) of the chest^[9]^. Although late detection of secondary lung cancer has a very poor prognosis, early radiation therapy, chemotherapy, and metastasectomy have been shown to significantly increase patient survival^[10,11]^. Therefore, for the optimal sequence and timing of local interventions, it is imperative to improve early detection of metastatic disease.

In order to improve the clinical detection of secondary lung cancers and to complement current imaging strategies, recent research efforts have led to the development of robust non-symptom-driven molecular screening assays. These assays have been designed to detect circulating primary tumor bioproducts such as circulating tumor cells (CTCs), circulating tumor DNA (ctDNA), circulating RNA transcripts (i.e., circular RNAs, messenger RNAs (mRNA), microRNAs (miRNA) and long non-coding RNAs), and more recently circulating tumor extracellular vesicles (EVs)^[12-18]^. Although the detection of CTCs has been used prognostically, it is not foolproof and a significant subset of patients who develop metastatic disease are not identified using this approach^[19-22]^. Although there are known biomarkers for the detection of specific cancers (e.g., AFP+CEA+CA125 for primary breast cancer or PSA for prostate cancer), there are currently no blood-based biomarker assays that can predict the development of secondary lung cancer^[23]^. Even though direct airway collection strategies [i.e., nasal epithelial brushing, sputum, bronchial brushing, bronchioalveolar lavage (BAL), and exhaled breath condensate (EBC)] are being investigated for the detection of primary lung cancer, they have not been evaluated for the detection of secondary lung cancer^[24-32]^.

The growing field of breath biopsy (i.e., the collection and analysis of exhaled breath) is showing great promise for the detection of lung tumors^[32-34]^. Indeed, the analysis of volatile organic compounds (VOCs) (i.e., ammonia, nitric oxide, hydrogen sulfide, acetone, aldehyde, methane, ethane, propane, and carbon dioxide)^[35,36]^ and non-volatile organic compounds (non-VOCs) (e.g., urea, amino-acids, RNA, DNA, proteins, lipids, surfactants) has been shown to reflect the metabolic and biologic repercussions of lung tumors^[37,38]^. Ongoing studies on VOCs are aimed at identifying metabolic changes associated with disease, whereas non-VOC studies are aimed at identifying and measuring biological compounds originating from tumor cells^[39,40]^. The collection of VOCs requires air-tight equipment for instant electronic analysis of exhaled gasses, whereas non-VOCs can be collected by the condensation of exhaled vapors to provide an exhaled breath condensate (EBC) biofluid, which can be stored and subsequently analyzed using different types of molecular assays (i.e., NGS, qPCR, methylation assays, *etc.*)^[41,42]^. Studies that have been conducted on EBC have revealed that it contains miRNAs, including those deregulated and released by tumors, with unique expression ratios that may be quantifiable and, in turn, allow detection of lung tumors^[43-47]^. Interestingly, recent investigations have also revealed that EBC contains extracellular vesicles (EVs), whose stably packaged miRNA cargoes can be analyzed and that may also help improve the detection of lung diseases^[48-50]^.

It is well documented that miRNAs are involved in the regulation of all biological processes^[51,52]^, and a large body of research has demonstrated that the deregulation of miRNA expression is associated with the initiation, development, and metastatic dissemination of human tumor cells^[53-58]^. Recent studies have also shown that miRNA profiles of tumor cells can provide both diagnostic and prognostic information on tumor progression^[59-62]^. Importantly, tumor cells can exchange miRNAs with both neighboring and distant cells via EVs. The molecular cargoes of tumor EVs, particularly miRNAs, have been shown to play important roles in the transcriptomic reprogramming of target cells^[63-65]^. For example, tumor EV miRNAs of breast, lung, and other types of human tumors have been associated with the modulation of angiogenesis^[60,65]^, cellular proliferation^[66-68]^, immune response^[69,70]^, and the establishment of distal pre-metastatic niches^[71-75]^.

EVs represent a large family of robust phospholipid bi-layered membrane-bound nanoparticles that are secreted by all human cells and can diffuse within tissues, circulate in the bloodstream, and be found in all biofluids^[76,77]^. EVs share common surface protein markers (i.e., CD9, CD81, CD83, Flotilin, *etc.*), as well as unique surface protein markers acquired from their cell of origin, which can be targeted by antibodies in molecular assays designed for their purification^[78-83]^. Studies of the miRNA content of circulating tumor EVs, specifically those purified from the circulation or those from other biofluids, have identified unique profiles, which can be associated with their tumor cells of origin^[84]^. Therefore, it is well perceived that the isolation of tumor EVs from biofluids, followed by the analysis of their miRNA cargos, has the potential to enable the development of non-invasive tumor detection assays for diagnostic and prognostic applications^[85-91]^.

In this proof-of-principle study, we sought to explore the potential of utilizing exhaled miRNAs for non-invasive detection of secondary lung cancer in orthotopic animal models. For these analyses, we chose to inoculate a highly metastatic breast cancer cell line that has been well documented to rapidly establish significant pathological lung tumor burden in athymic nude mice, which provided an adequate model to test the collection and analysis of exhaled breath condensates^[92-95]^.

## MATERIALS AND METHODS

### Cell culture

MDA-MB-231 subline 3475 triple-negative breast cancer cells were selected because of their aggressive and targeted lung tumor growth. MDA-MB-231 subline 3475 cells expressing both TdTomato-Luc and CD63-GFP were cultured in a standard growth media comprised of Dulbecco’s Modified Eagles Medium (DMEM) supplemented with 10% EV depleted fetal bovine serum (FBS) and 1% Penicillin Streptomycin. Cells were maintained at an atmosphere of 37 °C and a humidity of 5% CO2 and regularly subcultured once confluency of 70%-80% was reached. Upon reaching 80% confluency, cells were split with fresh media and allowed to undergo two rounds of passaging after cracking vials.

### Animals

All animal husbandry and procedures involving mice in this study were conducted under the Center for Discovery and Innovation IACUC approved protocol (#288.00) in an Association for Assessment and Accreditation of Laboratory Animal Care International (AAALAC) accredited research animal facility in accordance with all NIH guidelines for the use and care of experimental animals.

### Tumor inoculations for lung metastasis

On the day of tumor inoculations, TdTomato-Luc+/CD63-GFP + MDA-MB-231 subline 3475 cells were trypsinized and counted prior to being resuspended in warm 1x sterile PBS at a concentration of 1 × 10^6^ cells per 200 μL. Immediately after cell preparations, a heating pad was placed under one side of the cage to “pre-warm” and dilate veins of athymic BALB/C mice, animals were restrained, and the lateral tail vein was located. The needle was inserted parallel into the vein and cells slowly injected. Any bleeding at the injection site was stopped by applying gentle compression and animals were returned to their cage and monitored.

### In vivo bioluminescence imaging

Animals were anesthetized by isoflurane inhalation prior to receiving an intraperitoneal (I.P.) injection of D-luciferin (150 mg/kg). 15 min after D-luciferin administration, animals were placed onto the warming pad in the imaging box of an IVIS instrument, oriented so that tumors located in the lungs were well within the imaging area. Anterior images were acquired using the auto-exposure feature. Animals were imaged once a week for the duration of the study to: (i) determine the site of cancer cell growth; and (ii) monitor tumor burden.

### Animal condensate collection using the RC3 dual mouse chamber

Animal condensate collection was achieved by placing two mice together into a sterile glass RC3 respirometer chamber (Sable Systems International) attached to a SS4 flow pump/meter set to a rate of ~2.0 mL/min. The flow pump/meter sets the rate of inlet air into the chamber, with the ~2.0 mL/min setting being the recommended flow rate needed to allow for enough air flow into the chamber to prevent mice from suffocating during the collection period. The RC3 respiratory chamber is large enough to easily allow two mice to be placed in the chamber and does not lead to significant restraint (length = 10 and diameter = 3), as mice still have freedom to move around with normal postural movement (i.e., walk and turn around freely). Mice were kept in the chamber for one h to allow for adequate volumes of condensate to be collected. EBC collection was performed every week for the duration of this 16-week study.

### Exhaled breath condensate collection using a single mouse nose-mouth device

To collect exhaled breath condensate (EBC) from single mice without the risk of contamination from urine, skin and feces, we modified the design and setup of the single breath collection device described by Liu *et al.* in 2019^[96]^. For this method of EBC collection, single mice were placed into the modified mouse restrainer designed to expose only the nose-mouth of mice, enabling uncontaminated collection of EBC. The design of our single-mouse EBC collection device involves placing the mice into a restrainer that does not allow the mice to turn around so that maximum EBC volumes can be obtained. Since the use of full body restraint of animals can lead to enhanced stress, we designed the restraint in such a way that the head and torso of the animals were held within a dark chamber, allowing for animals to be within a dark enclosed space to reduce anxiety throughout the collection time-period (IACUC approved). Our single-mouse EBC collection device utilizes the same flow pump/meter described above for the collection of EBC from a dual mouse chamber, and the rate of inlet air into the chamber will be set to ~2.0 mL/min. Additionally, in order to ensure one-way air flow, a second flow meter will be attached to the other end of our collection (condensation) chamber and will be set to a flow rate of ~0.2 mL/min. Since our single collection device was smaller in size to ensure the collection of EBC only from the nose-mouse of animals, these collections provided smaller volumes of EBC and were performed for 2 h three times a week. The IACUC committee identified the duration and frequency of our collection acceptable to prevent animal stress throughout the duration of this study (24 weeks).

### Lung tissue collection and H&E staining

At the end of the study period, mice were sacrificed and lungs were perfused and inflated for tumor histological examination by inserting a 3 mL syringe with a 22 g needle attached into the trachea and slowly inflating the lungs with 10% formalin at a rate of approximately 200 μL/sec until the lungs have fully inflated. Following inflation, the trachea was severed, and the lungs removed from the respiratory cavity and placed into a tube containing 10% buffered formalin and were fixed for 24 h. Following fixation, lungs were processed by histological sectioning and H&E staining at the Histology & Comparative Pathology core facility at the Albert Einstein College of Medicine, Bronx, NY.

### Spectradyne microfluidic resistive pulse sensing

Particle size distribution of exhaled EVs isolated from mouse EBC was performed using microfluidic resistive pulse sensing (MRPS) measurements on a nCS1 instrument (Spectradyne LLC, Signal Hill, CA). Initially, the microfluidic system was primed using a solution of 0.2 µm filtered PBS containing 1% Tween 20 (v/v). For each purified exhaled EV sample, 2 μL was loaded onto a C-400 cartridge (i.e., analysis of particles between 65 and 400 nm), and the instrument pressure and voltage parameters were automatically determined using the instrument software. Acquisition of data from > 6,000 particle detection events was collected for each sample, and all data were combined into a single stats file and using the nCS1 Data Viewer software, peak filters and background subtraction were applied, according to the manufacturer’s recommendations. Peak filters set were (i) transit time < 60 μs; (ii) diameter > 65 nm, and signal-to-noise ratio (S/N) > 10. Additionally, combined stats files were analyzed for size distribution and particle concentration and peak-filtered CSD graphs were generated.

### EV-CATCHER isolation of the extracellular vesicles

The isolation of LM-3475 EVs *in vitro* and exhaled EVs from EBC collected *in vivo* was performed using the EV-CATCHER isolation protocol described by Mitchell *et al*. in 2021, customized with either human-specific CD63 or mouse-specific anti-CD63 capture antibodies^[97]^. Briefly, equimolar amounts of 5’-Azide modified and 3’-Biotin modified oligonucleotides (Integrated DNA Technologies) were annealed in 1x RNA annealing buffer, prior to separation on a 15% non-denaturing polyacrylamide (PAGE) gel. The double-stranded (ds) DNA product was visualized using SYBR® Gold™ (ThermoFisher, cat#S11494), excised, crushed, resuspended in 400 mM NaCl and placed on a thermomixer set to 4 °C and 1,100 RPM overnight. The solution was filtered, and the dsDNA linker was purified using the QIAEX® II gel extraction kit (Qiagen, cat#20021) according to the manufacturer’s instructions. Capture antibodies (1 mg/mL) were activated using 5 µL of freshly prepared 4 mM DBCO-NHS ester (Lumiprobe, cat#94720) and incubated for 30 min at room temperature (RT) in the dark; reactions were stopped by adding 2.5 µL of 1M Tris-Cl (pH 8.0) at RT for 5 min in the dark. DBCO-activated antibodies were desalted using Zeba desalting columns (ThermoFisher, cat#89882), and quantified on a Nanodrop 2000 instrument prior to the preparation of antibody-dsDNA (Ab-dsDNA) stock solutions. Ab-dsDNA conjugates were then bound to streptavidin-coated 96-well plates (Pierce, cat#15120) and wells were washed three times with cold 1x PBS solution, prior to the addition of RNase-A (12.5 μg/mL) treated samples (100 μL). Plates were sealed using microAMP optical adhesive film (Applied Biosystems, cat#4311971) and placed on a shaker at 300 RPM at 4 °C, O/N. Samples were carefully removed, and wells were washed 3 times with cold 1 × PBS, and 100 μL of freshly prepared uracil glycosylase (UNG) enzyme (ThermoFisher, cat#EN0362) in 1 × PBS [1 × UNG buffer 200 mM Tris-Cl (pH 8.0), 10 mM EDTA, and 100 mM NaCl], with 1 unit of enzyme, was added to each well. Plates were incubated at 37 °C for 2 h on a shaker at 300 RPM for UNG digestion of the dsDNA linker, and isolated EVs were collected for downstream analyses. For evaluation of the anti-human anti-CD63 EV-CATCHER assay, we conducted *in vitro* EV uptake experiments using EVs produced by LM-3475 cells transduced with a pCT-CD63-GFP lentivirus. A total of 180 mL of media was ultracentrifuged and the pelleted EVs were quantified using a Spectradyne nCS1 instrument. Half of the isolated EVs were subjected to the anti-human anti-CD63 EV-CATCHER assay and both EV-CATCHER and ultracentrifugation isolated EVs were used for *in vitro* uptake analyses, where non-transduced LM-3475 cells were treated with 1 × 10^10^ isolated EVs and confocal microscopy was performed to evaluate EV uptake, by measure of GFP fluorescence. All other EV-CATCHER purifications were conducted using mouse EBC collected *in vivo* using our EBC collection systems.

### Transmission electron microscopy

Transmission electron microscopy (TEM) of exhaled EVs purified by ultracentrifugation and the EV-CATCHER assay, obtained from 3 mL of mouse EBC (i.e., collected from six mice over a period of three weeks), was performed at the Analytical Imaging Facility at the Albert Einstein College of Medicine, Bronx, NY.

### ONi super-resolution nanoimaging

Exhaled EVs purified from mouse EBC by ultracentrifugation of 1 ml of EBC collected with our v2.0 system from lung tumor-bearing female mice (weeks 19-22) and from 3 mL of EBC from control female mice (weeks 19-22), were processed for nanoimaging on the highly sensitive ONi super-resolution Nanoimager using the ONi human EV Profiler kit v2.0 customized for the capture and assessment of EVs immobilized using their proprietary S4 capture molecule which binds phosphatidylserine present on all EVs. Mouse EVs ultracentrifuged from control female mice were only tested as a negative control, as ONi does not manufacture a mouse EV profiler kit. Human EV capture and staining was performed according to manufacturer protocol, and image acquisition on the ONi super-resolution Nanoimager was performed in the NimOS Light program with a 640 dichroic split using the following parameters: 640 nm laser set to 20%-30% laser power, the 560 nm laser at 35% laser power, and the 473/488 nm laser set to 70% laser power. Technical support from ONi provided information that during image acquisition, the fluorescent wavelength of TdTomato could not be excited and captured by either of the three pre-calibrated lasers and that the analysis of potential GFP signal was not detected in any of our raw data. The number of runs (frames) for all laser lines was set to 1,000 and all image analyses were performed using CODI software.

### Small-RNA extractions

Small-RNA extractions from exhaled EVs were performed using the miRNeasy Serum/Plasma kit (Qiagen, Cat#217184) according to the manufacturer’s instructions, with some modifications to improve total RNA yield. Briefly, QIAzol was added to 100 µL of purified exh-EVs, vortexed and incubated at RT for 3 min, after which chloroform was added to each sample. Samples were vortexed again and incubated at RT for 3 min. Samples were then centrifuged at 12,000 × *g*, at 4 °C for 15 min, and the upper aqueous phase was carefully removed and transferred into new siliconized 1.5 mL Eppendorf tubes, to which 100% ethanol and 2 µL of miRNA-Seq 19 nt/24 nt (1.5 ng + 1.5 ng) size marker required for NGS library preparations was added to each sample. Samples were incubated on ice for 40 min prior to undergoing column purification, where each sample was passed twice through RNeasy MinElute columns, followed by a working solution of RPE wash buffer, and finally ice-cold 80% ethanol. Columns were spun to remove residual ethanol, and total RNA was eluted with 50 µL of RNase-free water and samples were speed-vacuumed to 9.5 µL prior to small-RNA sequencing.

### Small-RNA cDNA library preparations

Small-RNA sequencing from EVs purified from mouse exhaled breath condensate (EBC) was performed using the cDNA library preparation protocol described by Loudig *et al*.^[98]^, with modifications for low input RNA from purified small EVs^[97]^. In brief, small-RNA cDNA libraries were prepared using total RNA recovered from condensates, whole EBC, or exhaled EVs purified with EV-CATCHER from EBC. 18 RNA samples underwent individual ligations using truncated K227Q T4 RNA Ligase 2 (New England Biolabs, cat#M0351L) for barcoding with 3’ adapters. The next day, ligations were heat inactivated at 90 °C for 1 min, combined, precipitated on ice, and centrifuged for 1 h at 14,000 RPM, at 4 °C. The pellet was dried, resuspended, and ligated miRNAs were size-selected on a 15% Urea-PAGE gel, excised, and incubated in 400 mM NaCl O/N at 4 °C, at 1,100 RPM on a thermomixer. The next day, the solution was filtered, precipitated, and a RNA pellet was obtained by centrifugation at 14,000 RPM for 1 h at 4 °C. The 5’ adapter was added to the resuspended pellet using T4 RNA Ligase 1 (New England Biolabs, cat#M0204L) for 1 h at 37 °C. The ligated product was separated on a 12% Urea-PAGE gel, size-selected, and excised; the gel fragment was crushed, resuspended in 300 mM NaCl solution with 1 mL 100 M 3’ PCR primer, and incubated O/N on a thermomixer at 1,100 RPM at 4 °C. The next day, the solution was filtered, precipitated with 100% ethanol, incubated on ice for 1 h, and pelleted by centrifugation for 1 h at 4 °C. The RNA pellet was resuspended, and underwent reverse transcription using SuperScript® III Reverse Transcriptase (ThermoFisher, cat#18080-093) at 50 °C for 30 min. The reaction was deactivated at 95 °C for 1 min, and a pilot PCR reaction was set up to identify the ideal amplification cycle. Large-scale PCR reactions were set up, combined, precipitated, digested with PmeI for removal of size markers, and separated on a 2.5% gel. The 100 nucleotide PCR library product was excised, purified with QIAquick Gel Extraction Kit (Qiagen, cat#28704), and quantified. cDNA libraries were then sequenced (single-read 50 cycles) on a HiSeq2500 Sequencing System, after which FASTQ files containing raw sequencing data were processed for adapter trimming and small-RNA alignment to the hg-19 genome.

### miRNA data analysis

Raw FASTQ data files obtained on an Illumina HiSeq2500 sequencer were processed using the RNAworld server from the Tuschl Laboratory at the Rockefeller University, including adapter trimming and read alignments and annotation. MiRNA counts were exported to spreadsheets for data analysis. Statistical analyses of miRNA counts were performed using dedicated Bioconductor packages in the R platform, as detailed below. Heat maps were generated from transformed counts using the “NMF” package (aheatmap function). Differential expression was assessed using “DESeq2” and “edgeR”. Differential expression models included a batch variable (library) to reduce batch biases. To maximize the discrimination ability of miRNA, we computed a score for each sample (“miRNA score”^[99]^), assembled by summing the standardized levels (z-values) of all significantly upregulated miRNAs, and the negative of the z-values of all significantly downregulated miRNAs.

### Proteomic data analysis

We conducted proteomic analyses on EBC samples collected with our v1.0 system and separately combined from the EBC of 6 control mice (i.e., 3 males and 3 females) and the EBC of 6 lung tumor-bearing mice (i.e., 3 males and 3 females). We estimated the total protein content of each of these two EBC samples (i.e., control and lung tumor-bearing samples) to be 53 ng and 76 ng, respectively. We also conducted proteomic analyses on EBC samples collected with our v2.0 system and separately combined EBC from 3 female control mice and from 3 female lung tumor-bearing mice and estimated the total protein content to be 29 ng and 38 ng, respectively. Proteins present in mouse EBC were analyzed by a workflow integrating suspension trapping (S-Trap)-based sample processing and data-independent acquisition mass spectrometry (DIA-MS) was recently described for the analysis of low input EV proteins^[100]^. In brief, proteins were extracted with 5% SDS, reduced with DTT, alkylated with iodoacetamide, and then digested on a S-Trap column (ProtiFi, LLC) with sequencing-grade trypsin/Lys-C (Promega). The resulting peptides were analyzed with a nanoAcquity UPLC system (Waters) coupling with an Orbitrap Fusion Lumos mass spectrometer (Thermo Fisher) in DIA mode, with parameters similar to those described previously^[100]^. The DIA data files were processed by Spectronaut (Biognosys) with default settings, and the peptides identified were aligned against mouse and human protein databases. For proteins identified from EBC collected with our v1.0 system, we identified a total of 448 proteins and a total of 333 proteins from EBC collected with our v2.0 system. Differential expression analyses [i.e., Ratio of lung tumor-bearing protein reads/control protein reads from condensates (v1.0 system) and EBC (v2.0 system)] of proteins commonly detected between lung tumor-bearing and control mouse biofluids [i.e., condensates (v1.0 system) and EBC (v2.0 system)] are displayed as heat maps.

## RESULTS

### Orthotopic tumor-bearing mouse model of secondary lung cancer

**Figure 1:**
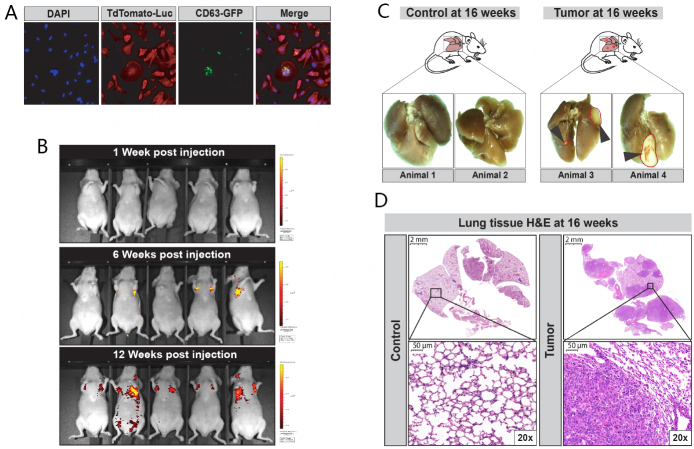
Establishment of the mouse model of human secondary lung cancer. (A) MDA-MB-231 subline 3475 cell line stably transduced with lentiviral constructs for expression of TdTomato-luciferase (pUltraChili-Luc) and pCT-CD63-GFP. Confocal imaging of a single clone co-expressing both TdTomato and GFP, sorted by FACS [See Supplementary Figure 1], with DAPI staining to locate cellular nuclei (Blue color, left panel) and Td-tomato red protein expression (Red colored cells, second panel from the left) and the CD63-GFP protein on extracellular vesicles (EVs, Green dots, third panel from the left), with a Merged image all staining and fluorescent imaging on the right panel. CD63-GFP detection shows that cells at different mitotic stages produced different amounts of EVs as observable by the differential detection of GFP protein. The central dividing cell displays the highest amount of observable EVs; (B) Bioluminescent *in vivo* imaging of tumor-bearing mice at weeks 0, 6, and 12 after inoculation of TdTomato-Luc+/CD63-GFP+ MDA-MB-231 subline 3475 cells. Bioluminescence intensity is indicated by means of radiant efficiency (photons/sec/cm^2^/sr) scale bars, with red being the most intense (See scale bar); (C) Representative formalin-fixed whole lung tissue images collected from two healthy mice (control; left) and two TdTomato-Luc+/CD63-GFP+ MDA-MB-231 subline 3475 inoculated lung tumor-bearing mice (case; right); (D) Representative images of Hematoxylin and Eosin (H&E) stained 5 mm tissue sections of lungs harvested from one control (left panels; 0 × and 20 × magnifications) and one lung tumor-bearing animal (right panels; 0 × and 20 × magnifications). The image right panel from one lung tumor-bearing animal shows extensive infiltration of metastatic carcinoma legions with a few rare immature lymphocytes seen interspersed.

The goal of our proof-of-principle study was to determine whether secondary lung cancer can be detected non-invasively at an early stage through the analysis of exhaled breath condensates. Thus, we selected an aggressive human metastatic breast cancer cell line (MDA-MB-231 subline 3475), which was developed by the group of Dr. Massagué, that, when delivered via tail vein injection, migrates and colonizes the lungs, rapidly forming expanding lung tumor foci within 15 weeks^[101]^. In previous studies, we demonstrated that these cells preferentially colonized the lungs of athymic BALB/C mice and led to a heavy tumor burden within 16 weeks^[102,103]^. Prior to conducting our *in vivo* analyses, we stably transduced these cells with the pUltraChili-Luc construct (TdTomato-Luc; 9947bp) [Supplementary Figure 1], as its luciferase production enables reduction of D-luciferin that is injected intraperitoneally (150 mg/kg) in anesthetized animals for release of bioluminescence and *in vivo* imaging of tumor growth*.* We also transduced these cells with the pCT-CD63-GFP Cyto-Tracer lentivirus (i.e., SBI, cat# CYTO120-VA-1, detailed in Supplementary Figure 1) to evaluate the purification and uptake of their extracellular vesicles *in vitro* [Supplementary Figure 2]. We used fluorescent activated cell sorting (FACS) to select for double-positive cells expressing both TdTomato-Luc [i.e., Supplementary Figure 1A] and CD63-GFP [i.e., Supplementary Figure 1B]. Single-cell expansion of double-positive LM-3475 clonal cells (i.e., high co-expression levels) was confirmed by confocal imaging in Figure 1A, using DAPI nuclei staining (blue; first panel), TdTomato-Luc (red protein, second panel) and CD63-GFP (green fluorescent protein; third panel). As cells were asynchronous and at different stages of mitosis, we observed different intensities of the GFP signal, indicating different production levels of CD63-GFP labeled EVs (Figure 1A, comparing GFP signal of central dividing cell with surrounding growing cells in third panel). Our double-positive LM-3475 clone was expanded *in vitro* and delivered via tail vein injection (i.e., 1 × 10^6^ LM-3475 cells) into male and female athymic BALB/C mice for our tumor-bearing animal group [Supplementary Figure 1C]. We conducted *in vivo* imaging weekly to monitor tumor localization and evaluate tumor burden in individual mice. We detected bioluminescent signal in the thoracic area of mice within 6 weeks after tail vein injection of double-positive LM-3475 cells [Figure 1B], which was consistent in all animals by 12 weeks [Figure 1B]. Our animals were sacrificed at 16 weeks, and we observed large macroscopic tumor lesions in the lungs of tumor-bearing animals [Figure 1C]. Our pathological evaluations revealed the presence of both microscopic tumor foci and large tumors within lung tissues [Figure 1D]. We did not observe significant differences in the growth or number of tumors between males and females.

### Evaluation of a whole mouse condensate collection system (Version 1.0)

**Figure 2:**
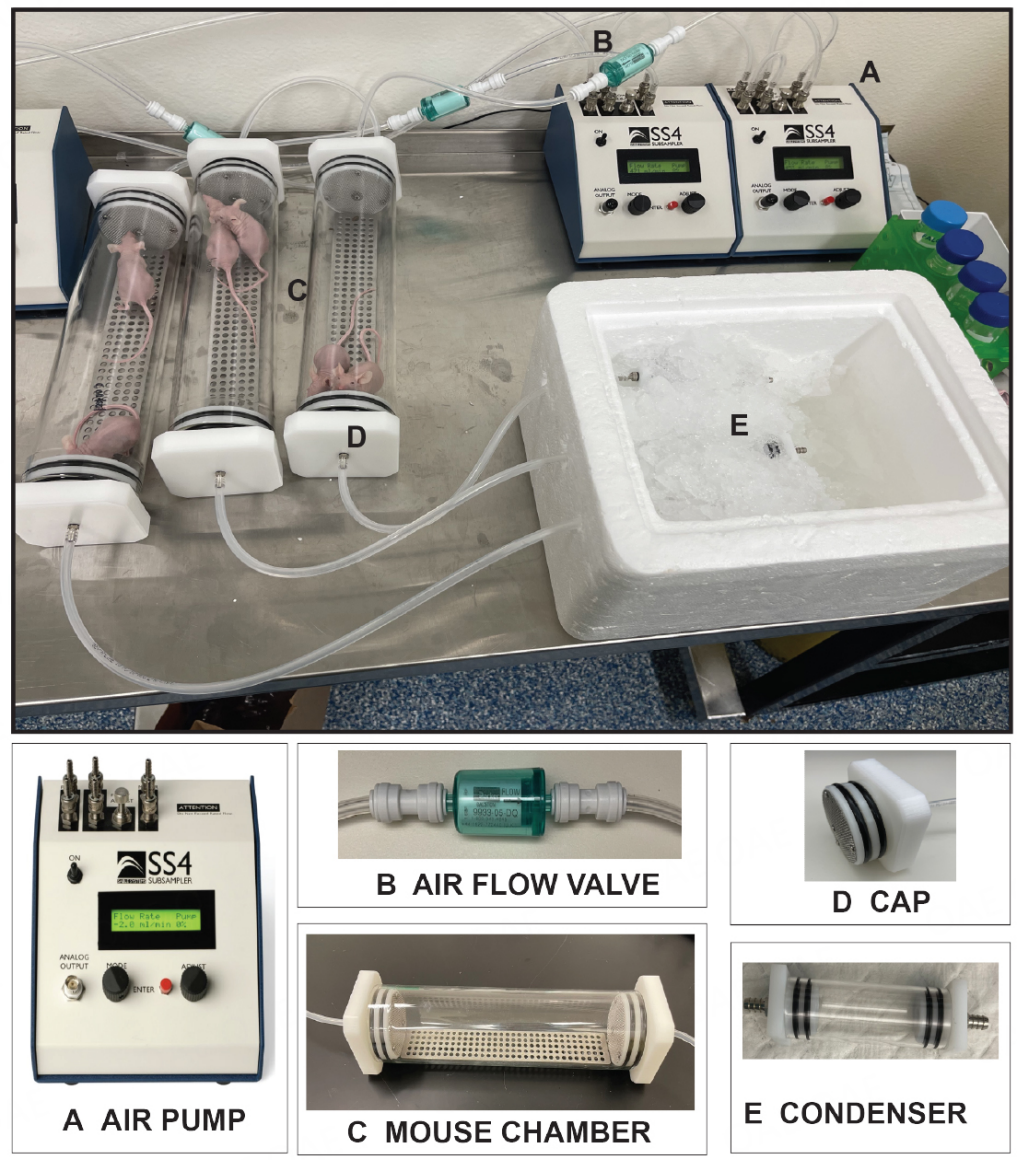
Whole mouse exhaled breath condensate (EBC) collection system version 1.0. For this system, EBC is collected from two unrestrained mice roaming in a sealed glass chamber, which contains a removable metal grate that allows animals to move freely with normal postural movement. Airflow throughout the system is maintained and directed towards a condenser. The components of this mouse EBC collection system include: (A) an air pump that controls airflow (2.0 mL/min) of compressed breathing-grade air that is transported through 1/4 inch plastic tubes; (B) a one-way Balston 0.01 mic 93% airflow filter that maintains air sterility; (C) a glass mouse chamber (containing two mice); that is (D) securely sealed on both ends by caps with gaskets; connected to (E) a glass condenser sealed on both ends by caps, which is placed on ice to allow for the collection of EBC. It is estimated that ~62.5 µL of EBC can be captured from two mice within one h of collection.

For these experiments, we sought to determine whether whole mouse condensates could be collected from unrestrained lung tumor-bearing and control athymic BALB/C mice. Utilizing the Sable Systems International (SSI) classic instrumentation line, we combined devices to enable collection of condensates in this air-tight system [Figure 2]. Using the SS4 pump (Figure 2A; flow rate of 2 mL per min), compressed breathing-grade air was circulated through a one-way Balston air flow filter (Figure 2B, to prevent air backflow) and through 1/4 inch tubes into the successive chambers [Figure 2C and E]. The first was the animal glass chamber (Figure 2C; accommodates up to two mice) that was sealed on both ends by caps with double gaskets to prevent air leakage [Figure 2D]. This chamber was fitted with a removable metal grate to allow mice to move freely and to allow urine and feces to drop in the lower section of the glass chamber. As the air was continually pushed through the mouse glass chamber, it was then directed towards a second small glass chamber (Figure 2E; the condenser), which was set on ice to enable condensation of animal aerosolized biofluids (condensates). This glass condenser was also sealed on both ends with tight-fitting endcaps equipped with double gaskets that prevented air leakage. For our experiments, we collected condensates from animal pairs of the same sex weekly for 16 weeks. Our weekly collections revealed that we could condense an average volume of ~62.5 µL of biofluid in each condenser within one h [i.e., average collected over 96 collections (3 animal pairs × 1 collection per week × 16 weeks × 2 groups)]. For our experiments, we collected three distinct condensates from dedicated animal pairs, separately from males (*n* = 6; 3 pairs) and females (*n* = 6; 3 pairs), both from our control (6 males and 6 females) and lung tumor-bearing (6 males and 6 females) animal groups.

### miRNA expression analyses of whole mouse condensates

**Figure 3:**
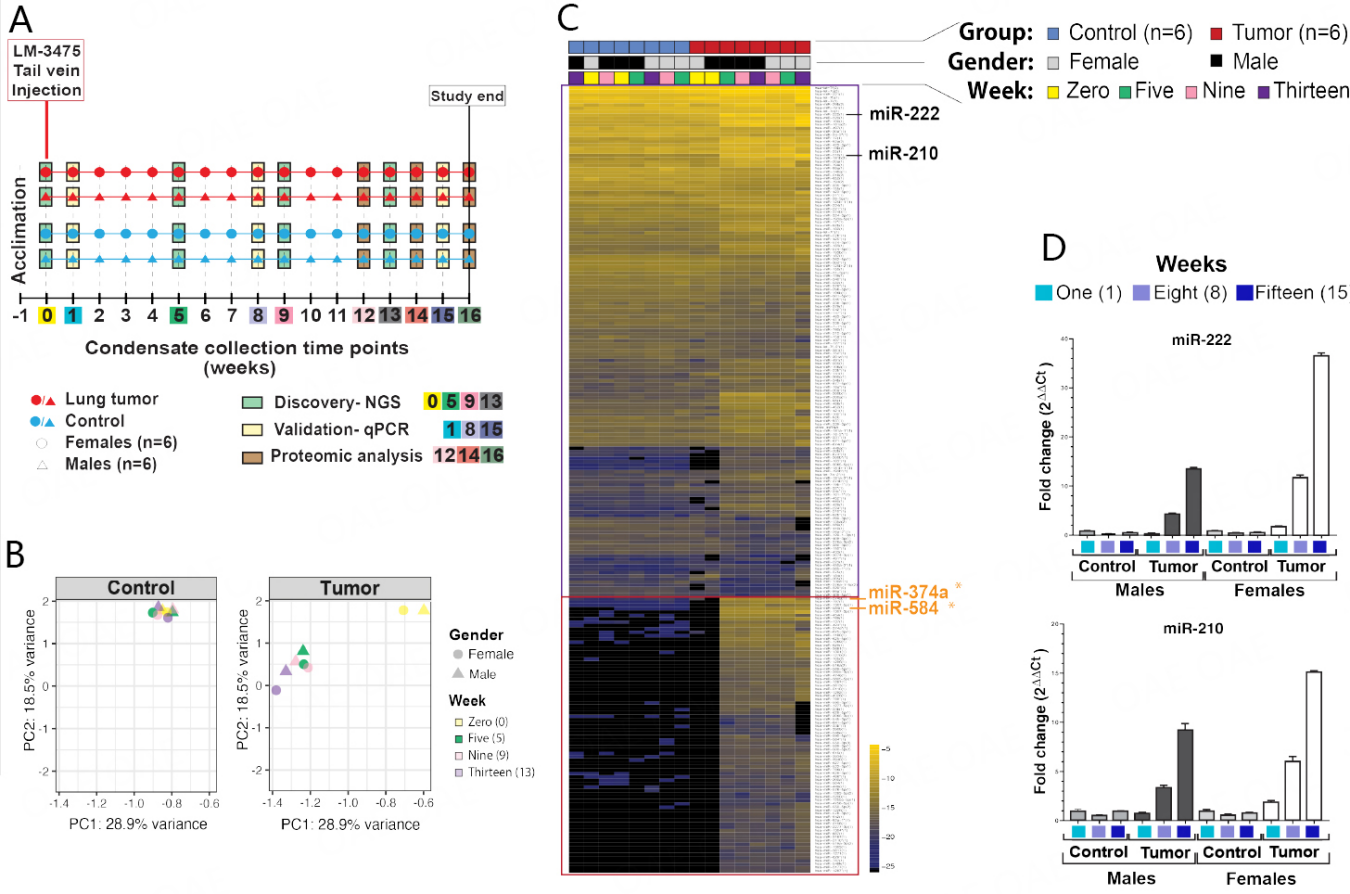
MicroRNA analysis of EBC collected from unrestrained animals. (A) Timeline of the weekly EBC collections from animal pairs, separated by sex (circles for females, triangles for males), between healthy control mice (blue) and lung tumor-bearing mice (red), for a period of 16 weeks. Discovery analyses were performed using total small-RNA extracted from EBC collected at weeks 0, 5, 9, and 13 using Next-Generation Sequencing (NGS). Validation analyses were conducted using total small-RNA extracted from EBC at weeks 1, 8, and 15 using quantitative reverse transcription PCR (RT-qPCR). Proteomic analyses were conducted on EBC collected and pooled for weeks 12, 14, and 16 from control and tumor-bearing mice groups; (B) PCA plots for miRNA expression of healthy control mice measured at weeks 0, 5, 9, and 13 for both females and males (top), and lung tumor-bearing mice at the same time points for both females and males (bottom); (C) Heatmap classification of the top 233 miRNAs detected by NGS using small RNAs extracted from EBC of healthy control (blue) and lung tumor-bearing (red) mice at weeks 0 (yellow), 5 (green), 9 (pink), and 13 (purple) for both females (grey) and males (black). The purple box highlights miRNAs commonly identified between condensates from control and lung tumor-bearing animals. The red box highlights miRNAs predominantly identified in condensates obtained from lung tumor-bearing animals. Two miRNAs, namely miR-374a and miR-584, which are bolded in orange text, are identified to be predominantly upregulated in condensates of lung tumor-bearing animals and are also identified to be upregulated in EBC obtained from lung tumor-bearing animals (system v2.0) displayed in Figure 7C and D. Taqman^©^ qPCR analyses of hsa-miR-222 and has-miR-210 using total small-RNA purified from EBC collected at weeks 1 (blue), 8 (light purple), and 15 (dark purple), separately for females and males, with data calculated using the 2^ΔΔCt^ formula between healthy mice (i.e., using week 1 Ct values as the reference), and lung tumor-bearing mice, both normalized to exogenous ath-miR-159a (100 pg) as an internal “housekeeping” control that was spiked in EBC before RNA extractions and qPCR analyses.

As we recently demonstrated that miRNAs are detectable in human EBC and that they may have diagnostic value for the detection of lung cancer^[43]^, we investigated whether microRNA expression analyses of our condensates could likewise distinguish mice with secondary lung tumors from control mice. We separately combined condensates from male pairs and from female pairs for each group and at each time point to enable RNA extractions from at least 100 µL biofluid. Then, using our optimized assays^[97,98]^, we conducted small-RNA next-generation sequencing (NGS) analyses of mouse condensates collected at weeks 0, 5, 9 and 13 from control females [Figure 3A, blue circles (*n* = 6)], control males [Figure 3A, blue triangle (*n* = 6)], and lung tumor-bearing females [Figure 3A, red circle (*n* = 6)], and lung tumor-bearing males [Figure 3A, red triangle (*n* = 6)]. Control male and female mice had received a mock injection of 1 × PBS at the same time as tumor-bearing male and female mice had received 1 × 10^6^ LM-3475 cells, which were delivered via tail vein injection at week 0, to eliminate experimental biases due to biofluid injections. Principle Component Analysis (PCA) plots revealed that the miRNA profiles captured from control male and female mice were consistent across the 16 weeks of collection [Figure 3B, control (blue)], but revealed differences between week 0 and weeks 5, 9, and 13 for lung tumor-bearing male and female mice [Figure 3B, tumor (red)]. Furthermore, we observed that the miRNA profiles of condensates collected at weeks 5, 9, and 13 from lung tumor-bearing mice clustered together, were consistent between males and females, but were highly different from those of male and female control mice [Figure 3C]. We noted that the miRNA profiles of condensates collected at week 0, following tail-vein injection of LM-3475 cells for lung tumor-bearing male and female mice, clustered together with those of control male and female mice [Figure 3C]. Overall, our miRNA profiles identified a set of miRNAs common between the control and tumor groups (Figure 3C, purple rectangle), and a set of tumor-associated miRNAs mostly detectable in the tumor group at weeks 5, 9 and 13, in both males and female mice (Figure 3C, red rectangle). Although we identified miRNA expression differences between the condensates of control and tumor groups, we could not fully determine whether all these miRNAs were of human (tumor cell) or mouse (mouse tissues) origin due to strong sequence homology between these two species.

As we sought to determine if we could conduct early and non-invasive detection of secondary lung tumors by analysis of miRNAs contained in condensate, we selected miR-222 and miR-210, which displayed high read counts by NGS, and had been reported in the literature for their increased expression in the circulation of women diagnosed with metastatic breast cancer^[104,105]^, to conduct our qPCR analyses [Figure 3D]. Our qPCR data confirmed their upregulated expression in condensates of lung tumor-bearing mice, but also revealed that their expression increased between weeks 1, 5, and 8 for both lung tumor-bearing males and females [Figure 3D]. We also noted that comparatively, the increase in expression of both miR-222 and miR-210 was higher in the combined condensates of lung tumor-bearing female mice than in those of male mice. Particularly, we observed that increased expression of both miR-222 and miR-210 was detectable in condensates of lung tumor-bearing female mice only one week after tail-vein injection of LM-3475 cells, but not in male mice. Our analyses demonstrated that miRNAs contained in condensates collected with our v1.0 system could discriminate control from lung tumor-bearing mice. Our data also suggested that metastatic human female breast cancer cells were detectable quicker in female than in male tumor-bearing mice.

### Proteomic analyses of whole condensates

**Figure 4:**
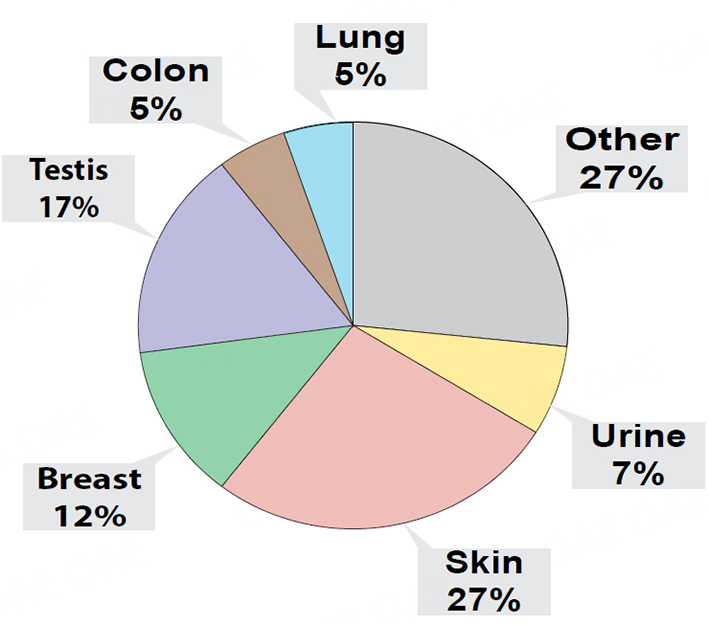
Proteomic analysis of EBC from healthy controls and lung tumor-bearing mice. Pie chart distribution of all 286 identified proteins in pooled EBC samples of control (*n* = 6) and lung tumor-bearing (*n* = 6) mice, stratified based on the preferential organ/tissue origin of each individual protein as informed by ProteinAtlas, and distributed as either from lung, skin, urine, breast, testis, colon, or undetermined tissue origins (i.e., Other).

Although our experiments indicated that condensates collected with our v1.0 system contained miRNAs that could discriminate control and lung tumor-bearing mice, we could not confirm that the condensed biofluids were solely obtained from exhaled breath. Indeed, upon the transfer of condensates to Eppendorf tubes, we could generally detect a smell of urine emanating from our condensates. Therefore, we sought to investigate the protein content of this biofluid to estimate tissue contribution, and used the S-trap-based sample processing coupled with DIA-MS proteomics approach, a workflow that we previously developed for the robust and ultra-sensitive proteomic analysis of low input proteins (as low as 5 ng input;^[100]^). We conducted these proteomic analyses on whole mouse condensates combined separately from 6 control (i.e., 3 male and 3 female mice with 53 ng of total protein input) and 6 lung tumor-bearing mice (i.e., 3 male and 3 female mice with 76 ng of total protein input), collected at weeks 12, 14, and 16 [Figure 4]. Our analyses identified a total of 448 proteins between condensates of control and lung tumor-bearing mice. We determined that 232 proteins were common to both control and lung tumor-bearing mouse condensates but that 216 were unique human proteins, which were only detected in condensates of lung tumor-bearing mice. When evaluating the 55 most differentially expressed proteins, common to both control and lung tumor-bearing mouse condensates (Supplementary Figure 3; aligning to both human and mouse protein sequences), we identified several upregulated proteins in the condensates of lung tumor-bearing mice, of which some have been correlated with pro-metastatic properties in previous studies (Supplementary Figure 3; Plectin, Gelsolin, Vimentin, β1 integrin, Integrin a6, α-enolase, S100A4)^[100,106-107]^. Due to the high sequence homology between human and mouse for these 55 proteins, mouse tissue or human tumor contribution could not be confirmed. However, when we conducted putative tissue origin analysis of the 232 proteins (i.e., based on prominent tissue expression determined in ProteinAtlas) common to both control and lung tumor-bearing mouse condensates, we determined that a large proportion could be categorized to other tissue origins than the lung or respiratory system (Figure 4, skin, urine, upper digestive tract, colon, testes, and breast).

### Evaluation of a nose/mouth targeted exhaled breath condensate collection system (Version 2.0)

**Figure 5:**
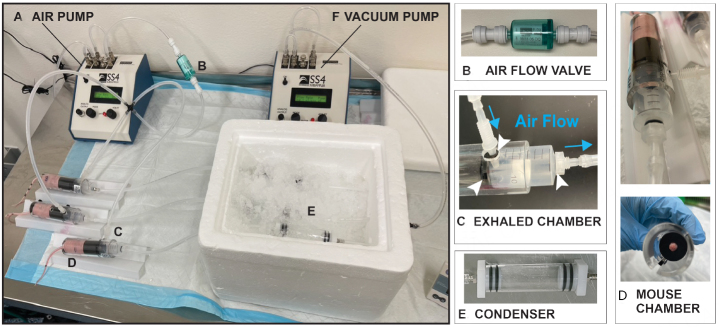
Nose and mouth EBC collection system v2.0 for restrained individual animals. The system designed and described here includes additional devices that enable air flow and direct collection of EBC from the nose and mouth of restrained mice. The system is composed of: (A) an air pump controlling airflow (2.0 mL/min) from an air tank transported by 1/4 inch tubes; (B) an airflow valve to maintain air directionality and sterility; (C) an exhaled chamber that is tightly connected with gaskets; to the (D) mouse immobilization chamber where the animal is restrained and through 1/4 inch tubes; towards (E) a glass condenser sealed on both ends by caps and sitting on ice for the accumulation of EBC droplets; which is connected to (E). a second air pump set up in vacuum mode to enhance air circulation through the entire system (i.e., set at 0.2 mL/min). This system allows for individual collection of ~29 µL EBC from restrained mice within 2 h.

Since our low output proteomic analyses suggested that whole animal collection of condensates introduced protein contaminants from tissue sources other than the lung, to improve our miRNA expression analyses, we sought to focus our collection on exhaled breath condensates. Thus, we improved the design of our collection system by including a mouse restraining device (i.e., as described by Liu *et al.*^[96]^) and by including an exhaled breath collection chamber to allow targeted capture of exhaled breath condensate (EBC) directly from the nose/mouth of individual mice [Figure 5]. Similarly to that of our v1.0 collection system, this system included an air pump (Figure 5A; set at 2 mL per minute) supplying compressed breathing-grade air, a one-way airflow Balston filter [Figure 5B] to enable unidirectional air flow, an exhaled breath collection chamber (Figure 5C; see blue arrows for airflow) that was sealed by a gasket at its connection at the end of the mouse restrainer [Figure 5D], and a sealed glass condenser [Figure 5E]. In order to enhance airflow, we added an air pump to gently pull air from the end of the condenser (Figure 5F; set at 0.2 mL per min). Importantly, we determined that within a 2-h collection window (i.e., the maximum allowed time by the internal IACUC committee), we could collect an average of ~29 µL of EBC from individual mice. Due to the collection of a lower EBC volume with our v2.0 collection system than with our v1.0 system (i.e., 63 µL per h), for experimental and feasibility reasons, we chose to analyze combined EBC samples (i.e., 3 control mice or 3 lung tumor-bearing mice) to reach a total working volume of 100 µL. Since we did not observe differences in the identity of the miRNAs differentially expressed in condensates collected from male and female lung tumor-bearing mice with our v1.0 system, but we observed increased detectability of miR-222 and miR-210 at earlier stages of disease (i.e., at week 1) in female mice compared to male mice, we chose to conduct our proof-of-principle miRNA NGS and qPCR analyses of EBC collected with our v2.0 EBC collection system from female mice only. This was further supported by the fact that the incidence of male breast cancer and its metastasis to the lung is extremely rare^[108]^, and that our cells were of female breast cancer origin.

### Detection of human tumor extracellular vesicles in mouse EBC

**Figure 6:**
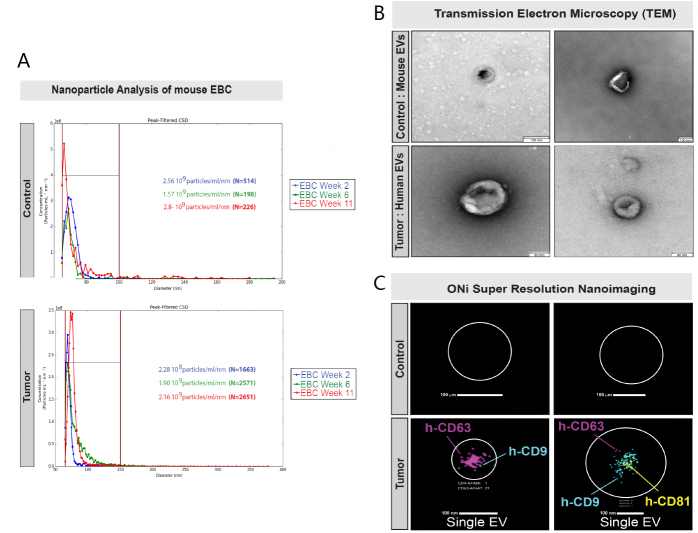
Purification and analysis of exhaled extracellular vesicles from mouse EBC. EBC collected directly from the nose and mouth of individual animals was evaluated for the presence of exhaled EVS. (A) EBC from healthy control (*n* = 3) and lung tumor-bearing (*n* = 3) mice collected at weeks 2, 6, and 11 was evaluated using the Spectradyne nCS1 nanoparticle analyzer, using C400 cartridges for the detection of nanoparticles between 65 and 400 nm; (B) EBC samples from 6 control (left; 3 mL) and 3 lung tumor-bearing (right; 1 mL) mice, collected over 4 weeks, were subjected to ultracentrifugation and the pellets analyzed by imaging using transmission electron microscopy (TEM); (C) EV pellets from healthy control (top panels) and from lung tumor-bearing mice were analyzed using Super-Resolution Microscopy (ONi instrument) using anti-human anti-CD63, anti-CD9, and anti-CD81 anti-tetraspanin antibodies to evaluate the size and identity exhaled EVs contained in the ultracentrifuged EBC pellets of control (top panels) and lung tumor-bearing mice (bottom panels).

As determined in previous studies, EBC collected with different types of condensing devices from humans has been found to contain EVs^[48-50]^. Thus, for this proof-of-principle study, we sought to determine whether EBC collected from the nose/mouth of restrained mice also contained EVs. Thus, we conducted nanoparticle analyses of whole EBC collected with the v2.0 EBC collection system from both control and lung tumor-bearing mice using our Spectradyne Nanoparticle Analyzer nCS1 instrument [Figure 6A]. Our analyses of EBC collected and pooled from three mice at weeks 2, 6, and 11 revealed that nanoparticles of 65-150 nm in diameter were detectable from both control (*n* > 57 nanoparticles, Figure 6A top graph) and lung tumor-bearing mice (*n* > 1,663 nanoparticles, Figure 6A bottom graph), but that they were present at much greater concentrations in the EBC of lung tumor-bearing female mice than control female mice. Additionally, we observed that the number of nanoparticles detectable in EBC of lung tumor-bearing mice between weeks 2 and 11 nearly doubled (Figure 6A; from 1,663 at week 2 to 2,651 particles at week 11). Next, we performed Transmission Electron Microscopy (TEM) on EVs ultracentrifuged from 1 ml of EBC collected and combined from 6 lung tumor-bearing female mice between weeks 19 and 22 using our v2.0 collection system (Figure 6B; bottom panels). Since our Spectradyne analyses showed that EVs from control mice were in much lower concentrations, we collected and combined 3 ml EBC obtained from 6 control female mice over a period of six weeks and then conducted ultracentrifugation and TEM analyses [Figure 6B]. EVs detectable from EBC of control and lung tumor-bearing female mice were ~80-100 nm in diameter [Figure 6]. Since our analyses indicated that EBC collected from lung tumor-bearing mice contained a higher number of nanoparticles, we sought to determine whether this increase was due to an output of human tumor EVs. Thus, we used the ONi human EV profiler kit to conduct super-resolution nanoimaging [Figure 6C]. With this kit, phosphatidylserine present in the membrane of EVs is bound onto the ONi platform with a proprietary S4 capture molecule, and they are then evaluated for presence of human CD9, CD63, and CD81 tetraspanin proteins by laser detection of their three individually fluorescently labeled anti-human antibodies [Figure 6C]. We note that the scanning wavelength of each of the three lasers used to detect these three fluorescent antibodies did not coincide with the fluorescent signal of TdTomato and that no signal was detected for GFP in any raw data files acquired from the imaging of exhaled EVs. Since a mouse EV profiler kit was not manufactured by ONi, our EV validation analyses were focused only on human EVs. Our analyses revealed that we could not detect human CD9, CD63, and CD81 from mouse EVs pelleted by ultracentrifugation from 3 mL of control mouse EBC and tested on the ONi platform (Figure 6C; upper panels). We also note that this volume was three-fold the volume of EBC collected and ultracentrifuged from lung tumor-bearing mice (i.e., 1 mL) and thus three-fold the input of mouse EVs present in EBC collected from lung tumor-bearing mice. Our analyses showed that we could individually detect human CD9, CD63, and CD81 proteins on the surface of EVs immobilized on the ONi platform, which were purified by ultracentrifugation of 1 mL EBC from lung tumor-bearing mice (Figure 6C, lower panels). We displayed scans of the ONi grids for all detectable EVs in Supplementary Figure 4. Our results suggest that the ONi human EV profiler kit could not capture or identify human CD9, CD63, or CD81 tetraspanins on EVs ultracentrifuged from the EBC of control mice (Figure 6C; top panels), but identified all three proteins on EVs ultracentrifuged from the EBC of lung tumor-bearing mice.

### miRNA analyses of EBC and exhaled EVs collected directly from nose/mouth

**Figure 7:**
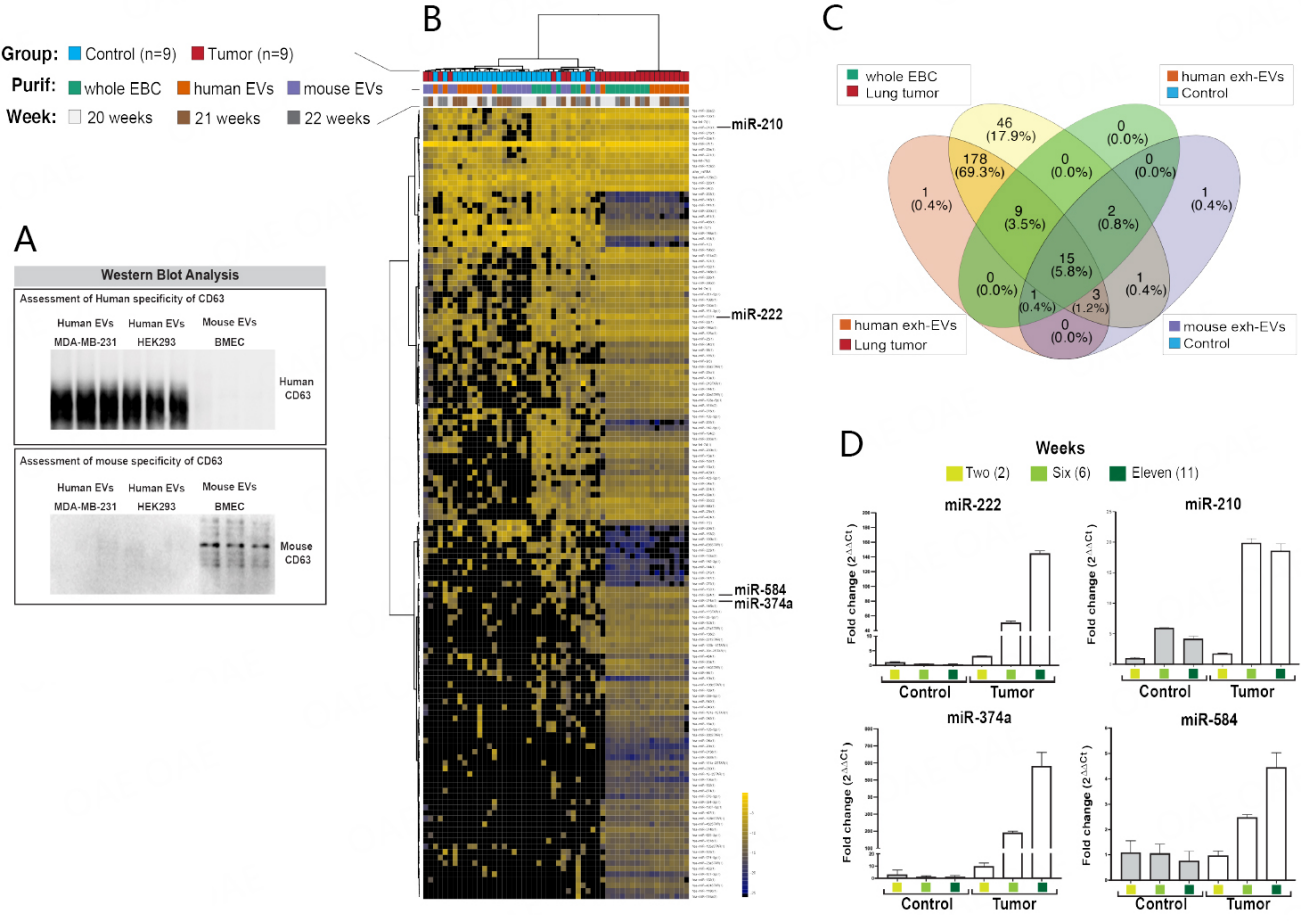
MiRNA analyses of EBC and exhaled EVs collected from control and lung tumor-bearing mice. (A) Western blot evaluation of anti-human and anti-mouse CD63 antibodies using EVs purified from tissue culture media of human breast cancer MDA-MB-231 cells, human kidney cancer HEK293 cells, and normal mouse bone marrow endothelial cells (BMECs); (B) Heatmap analysis of the top 142 most differentially detectable miRNAs between small-RNA extracted from whole EBC (green), and sequentially purified from human exhaled EVs using the anti-human anti-CD63 EV-CATCHER assay from whole EBC (orange), and mouse exhaled EVs using the anti-mouse anti-CD63 EV-CATCHER assay from the same whole EBC samples (purple), collected at weeks 21 (light grey), 22 (brown), and 23 (dark grey) from female control (blue) and lung tumor-bearing (red) mice detectable at study end (week 24). We conducted our analyses in triplicate (i.e., three repeats per RNA purification) on 9 control female mice and 9 lung tumor-bearing female mice. The EBC collected three times a week from the same 3 females was combined (~ 300 µL) to conduct the three different analyses (whole EBC, human exh-EVs, mouse exh-EVs) in triplicate [i.e., 3 sets of 3 EBC collections per control (*n* = 9) or lung tumor-bearing group (*n* = 9)]; (C) Venn Diagram displaying the overlap in the identity of the miRNAs detected between whole EBC of lung tumor-bearing mice (yellow), human exh-EVs in lung tumor-bearing mice (orange), mouse exh-EVs in control mice (purple), and human exh-EVs in controls mice (green, non-specific signal). The miRNAs that were selected for these analyses were detected by NGS but had at least 5 reads in 6 of the 9 samples analyzed and were reproducibly detected at least two of the three weeks (weeks 20, 21, and 22). The Venn diagram indicates that 21 miRNAs were non-specifically detected by use of the anti-human anti-CD63 EV-CATCHER assay with EBC of control mice and represented 9% of all selected miRNAs; (D) Small RNAs extracted from whole EBC samples collected at weeks 2, 6, and 11 were evaluated for expression of miR-222, miR-210, miR-374a, and miR-584 by Taqman^TM^ quantitative PCR analyses using the 2^ΔΔCt^ method to evaluate fold change by comparison to the control sample at week 2. All 4 miRNAs were selected because they were found to be upregulated in the whole EBC of lung tumor-bearing mice compared to control mice by NGS analyses. MiRNA analyses of EBC and exhaled EVs collected from control and lung tumor-bearing mice. (A) Western blot evaluation of anti-human and anti-mouse CD63 antibodies using EVs purified from tissue culture media of human breast cancer MDA-MB-231 cells, human kidney cancer HEK293 cells, and normal mouse bone marrow endothelial cells (BMECs); (B) Heatmap analysis of the top 142 most differentially detectable miRNAs between small-RNA extracted from whole EBC (green), and sequentially purified from human exhaled EVs using the anti-human anti-CD63 EV-CATCHER assay from whole EBC (orange), and mouse exhaled EVs using the anti-mouse anti-CD63 EV-CATCHER assay from the same whole EBC samples (purple), collected at weeks 21 (light grey), 22 (brown), and 23 (dark grey) from female control (blue) and lung tumor-bearing (red) mice detectable at study end (week 24). We conducted our analyses in triplicate (i.e., three repeats per RNA purification) on 9 control female mice and 9 lung tumor-bearing female mice. The EBC collected three times a week from the same 3 females was combined (~ 300 µL) to conduct the three different analyses (whole EBC, human exh-EVs, mouse exh-EVs) in triplicate [i.e., 3 sets of 3 EBC collections per control (*n* = 9) or lung tumor-bearing group (*n* = 9)]; (C) Venn Diagram displaying the overlap in the identity of the miRNAs detected between whole EBC of lung tumor-bearing mice (yellow), human exh-EVs in lung tumor-bearing mice (orange), mouse exh-EVs in control mice (purple), and human exh-EVs in controls mice (green, non-specific signal). The miRNAs that were selected for these analyses were detected by NGS but had at least 5 reads in 6 of the 9 samples analyzed and were reproducibly detected at least two of the three weeks (weeks 20, 21, and 22). The Venn diagram indicates that 21 miRNAs were non-specifically detected by use of the anti-human anti-CD63 EV-CATCHER assay with EBC of control mice and represented 9% of all selected miRNAs; (D) Small RNAs extracted from whole EBC samples collected at weeks 2, 6, and 11 were evaluated for expression of miR-222, miR-210, miR-374a, and miR-584 by Taqman^TM^ quantitative PCR analyses using the 2^ΔΔCt^ method to evaluate fold change by comparison to the control sample at week 2. All 4 miRNAs were selected because they were found to be upregulated in the whole EBC of lung tumor-bearing mice compared to control mice by NGS analyses.

Next, we sought to evaluate the utility of our EV-CATCHER assay customized for species-specific purification of exhaled EVs from mouse EBC^[97]^. Thus, we selected a human-specific and a mouse-specific anti-CD63 antibody to purify and conduct comparative miRNA expression analyses of human and mouse EVs contained in the EBC of control and lung tumor-bearing mice. We chose an anti-human anti-CD63 customized EV-CATCHER assay for the purification of human EVs from mouse EBC, as opposed to an anti-GFP antibody (i.e., CD63-GFP labeled EVs are produced by LM-3475 cells in Figure 1A), because we had demonstrated its high affinity for human EVs in a previous study^[97]^. Due to the limited availability of exhaled EVs, we initially tested the ability of our anti-human anti-CD63 EV-CATCHER assay to purify and release intact EVs produced by pCT-CD63-GFP transduced LM-3475 cells [Supplementary Figure 2] by measuring their uptake *in vitro*. When comparing the uptake of CD63-GFP EVs (i.e., produced by our LM-3475 cells) purified by ultracentrifugation or by our anti-human anti-CD63 EV-CATCHER by non-transduced LM-3475 cells, we similarly detected GFP labeled EVs inside treated cells using confocal microscopy [Supplementary Figure 2]. Prior to the purification of exhaled EVs, we also compared the species specificity of our anti-human and anti-mouse anti-CD63 antibodies by Western blot analyses of EVs ultracentrifuged from tissue culture-derived media of human MDA-MB-231 breast cancer cell, human HEK293 cells, and primary mouse bone marrow-derived endothelial cells (BMEC), which revealed high species-specific recognition of our anti-human and anti-mouse anti-CD63 antibodies [Figure 7A]. Considering the high tumor burden observed at 24 weeks (Supplementary Figure 5; see H&E), we chose to conduct our miRNA NGS analyses with EBC collected at weeks 20, 21, and 22 to ensure similar disease burden among the animals and high miRNA detectability. Prior to our analyses with NGS, we conducted species-specific sequential isolations of human and then mouse EVs from whole mouse EBC, using our respective anti-human and anti-mouse CD63-EV-CATCHER assays. Our NGS data of whole EBC (Figure 7B, blue and green squares) identified miRNAs unique to lung tumor-bearing animals compared to whole EBC of control mice (Figure 7B, red and green squares). Comparatively, when using our anti-human anti-CD63 EV-CATCHER to purify human tumor EVs from mouse EBC of lung tumor-bearing mice (Figure 7B, red and orange squares), we identified miRNAs, whose expression overlapped with those detected in whole EBC of lung tumor-bearing animals but not detected in control EBC (Figure 7B, blue and red squares). When using the anti-mouse anti-CD63 EV-CATCHER assay, we generated miRNA profiles (Figure 7B, blue and purple squares), which clustered closely to that of whole EBC from control mice (Figure 7B, blue and green squares). It is important to note that when using the anti-human anti-CD63 EV-CATCHER assay on EBC of control animals (Figure 7B, blue and orange squares) where no human EVs were anticipated to be present, due to the minimal cross-reactivity of the human anti-CD63 antibody against mouse CD63, we captured non-specific miRNA profiles that clustered between the miRNA profiles of whole EBC from control mice and mouse EVs purified with the anti-mouse anti-CD63 EV-CATCHER assay from control animals. As displayed in Supplementary Figure 6, the number of reads detected non-specifically (10^4^ reads) was 1,000-fold lower than the signal detected specifically with human exh-EVs purified from EBC of lung tumor-bearing mice (10^7^ reads). We noted that the non-specific capture of EVs using our anti-human anti-CD63 EV-CATCHER with EBC of control mice was ~10.5% (5.8% + 0.8% + 3.5% + 0.4%), or for 27 miRNAs that were unique to mouse whole EBC. However, we observed an overlap of 80.6% (69.3% + 3.5% + 5.8% + 1.2% + 0.4% + 0.4% or 207 miRNAs) of all 257 miRNAs reproducibly detected (i.e., with at least 5 reads in 6 out of 9 samples per group in 2 out of the three weeks) between whole EBC of lung tumor-bearing mice and human EVs purified from the EBC of lung tumor-bearing mice [Figure 7C]. These results confirmed that the bulk of differentially expressed miRNAs detectable in the EBC of lung tumor-bearing mice originates from human tumor exhaled EVs.

### Feasibility of detecting differently expressed miRNAs in EBC by qPCR analyses

Next, we sought to retrospectively validate our findings by evaluating EBC samples collected at earlier stages of disease (i.e., weeks 2, 6, and 11) and to determine whether we could detect metastatic lung tumors non-invasively by simple qPCR analysis of differentially expressed miRNAs identified by our NGS analyses [Figure 7D]. As similarly observed with our NGS data obtained from condensates collected with our v1.0 collection system, we also found miR-222 and miR-210 to be increased in expression in whole EBC of lung tumor-bearing animals collected with our v2.0 collection system, compared to EBC of control mice. However, we also identified two additional miRNAs (miR-374a and miR-584) that were mostly detectable by our NGS analyses in whole EBC and human tumor EVs of lung tumor-bearing animals but not detected in EBC of controls. When reanalyzing NGS data obtained with condensates collected from control and lung tumor-bearing mice with our v1.0 collection system (see Figure 3C; see miR-374a and miR-584), we noted that although both miRNAs had lower reads, they appeared also to be uniquely upregulated in condensates of lung tumor-bearing mice. When we conducted our comparative qPCR analyses of EBC collected from control and lung tumor-bearing female mice, we reproducibly validated the upregulated expression of all miRNAs in EBC of lung tumor-bearing mice. However, we found that miR-222 and miR-374a provided greater sensitivity for earlier detection of disease (Figure 7D; at week 2) than miR-210 and miR-584 (Figure 7D; at week 6). These results further confirmed that exhaled metastatic human lung tumor EVs provide molecular surrogates for non-invasive early detection of secondary lung tumors in mice.

### Proteomic analyses of EBC from individual animals

**Figure 8:**
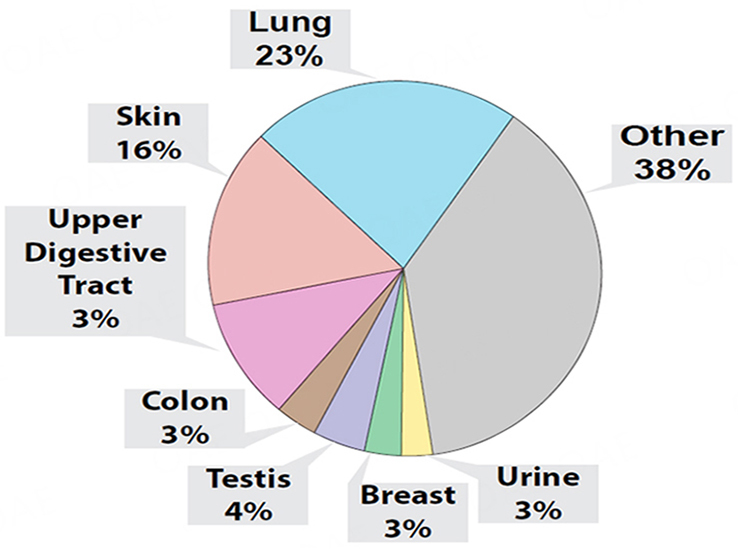
Proteomic analysis of EBC pooled from controls and lung tumor-bearing mice. Pie chart distribution of the 231 identified proteins stratified based on the preferential organ/tissue expression of each individual protein based on ProteinAtlas, and classified as either from lung, skin, urine, breast, testis, upper digestive tract, colon, and other undetermined tissue origins (i.e., Other).

Finally, using our EV proteomic analytical approach described above, we sought to confirm whether the protein content of EBC collected with our v2.0 system for nose/mouth collection from single animals allowed for a more targeted capture of biological material of lung tissue or respiratory tissue origin. Our EBC proteomic analyses identified a total of 286 proteins that were common to control female mice [*n* = 3; 29 ng total protein input for mass spectrometry (MS)] and lung tumor-bearing female mice (*n* = 3; 38 ng total protein input for MS). Due to high sequence homology between human and mouse proteins, we could not determine the human tumor or mouse tissue contribution. However, our proteomic analyses of EBC from lung tumor-bearing mice identified 47 additional human proteins (a total of 333 proteins). When selecting the top 60 most differentially expressed EBC proteins that were common to control and lung tumor-bearing EBC [Supplementary Figure 7], we confirmed detection of several proteins that had been correlated with pro-metastatic properties, which were also upregulated in condensates collected from lung tumor-bearing mice with our v1.0 system, but that displayed even greater differential expression in EBC collected with our v2.0 system (i.e., Vimentin, α-enolase, integrin B1, Plectin)^[100,106-107]^. Finally, when we analyzed the most likely tissue distribution/origin of the common proteins detected the EBC of both groups, by using prominent tissue expression as determined with ProteinAtlas, we found that our v2.0 collection system generally enabled the collection of EBC with a higher proportion of proteins of lung and respiratory tissue origin [Figure 8]. These proteomic analyses illustrate that adding a nose/mouth collection device improved performance of our EBC collections as we observed its enrichment with proteins of respiratory tissue origin.

## DISCUSSION

In this proof-of-principle study, we investigated whether the analysis of exhaled condensates collected from an orthotopic animal model of secondary lung cancer would enable the non-invasive detection of lung tumors. Considering that we and others have previously demonstrated that miRNAs can be reproducibly detected in human exhaled breath condensates^[43-47]^ and that they hold both diagnostic and prognostic potential for the detection of lung cancer, we developed collection systems and molecular assays to quantify them in mouse exhaled condensates.

In order to establish a baseline for the study of exhaled condensates from mice, we compared the collection and analysis of two types of condensates recovered from our mice using two different systems: one for the collection of condensates from unrestrained mice, freely roaming in a sealed glass chamber (v1.0 system), and one for the targeted capture of exhaled breath condensates directly from the nose/mouth of individually restrained animals (v2.0 system). Despite experimental and technical limitations due to the low volumes of condensate and EBC that were recovered with our v1.0 and v2.0 systems, respectively, we successfully conducted miRNA next-generation sequencing (NGS), qPCR validation, and used proteomic analyses to evaluate the condensates. Although our proteomic analyses yielded low outputs, we utilized our data to evaluate the potential origins of the proteins from condensate and EBC samples collected with our two different systems. Our results unequivocally demonstrated that human proteins can be specifically identified from condensates (i.e., 216 proteins) and EBC (i.e., 47 proteins) samples collected from lung tumor-bearing mice. However, when evaluating the putative tissue origin of proteins (*n* = 232) detected in condensates collected with our v1.0 system, which were common between control and lung tumor-bearing mice, we observed that a large proportion of them were associated with tissues/organs outside of the respiratory system (i.e., skin, urogenital (i.e., urine), reproductive (i.e., testis and breast), lower (i.e., the colon) and upper digestive tracts [i.e., esophagus and mouth)]. This suggested that the collection of condensates from free-roaming mice additionally gathered biological material from organs other than the respiratory system, which we anticipated would limit the discovery of exhaled human lung tumor proteins. Indeed, studies on metastatic breast cancer cell lines that were intentionally established to enhance metastatic colonization within the lungs upon inoculation into the circulatory system, demonstrated that some of these metastatic cells may colonize and expand as tumor foci in organs other than the lung^[101,109,110]^.

Thus, to improve the collection of exhaled lung condensates, we developed our v2.0 system, where EBC was directly captured from the nose/mouth of our mice. Although we noted a significant decrease in the volume of biofluid collected with our v2.0 system (i.e., from ~63 µL for unrestrained animal pairs/h to ~29 µL per single restrained animal per 2 h), we observed an increase in proteins originating from the respiratory system relative to the v1.0 system. As anticipated with lower volumes and thus lower amounts of condensed biological material (< 40 ng input for MS), our proteomic analyses of EBC identified a lower number of proteins but demonstrated that proportionally to those detectable with the v1.0 system, putative respiratory proteins were enriched in EBC collected directly from the nose/mouth of our animals with the v2.0 system. Although the v1.0 collection system generally appeared to provide a condensate that was less clean than EBC (i.e., proteins originating from urine, skin, semen, *etc.*), we believe that it holds value for the collection of condensates in animal studies investigating advanced lung diseases or injuries, where immobilization or animal manipulation is restricted due to shallow breathing and/or the risk of stress-related death. For example, when investigating the continuum of dose-dependent injury and/or recovery after inhalation of highly toxic chemicals, such as sulfur mustard, the analysis of lung injuries in rats can only be performed by collection and analysis of bronchoalveolar lavages (BAL) and by pathological evaluation of lung tissues at either scheduled study termination (i.e., 30 days) or via a serial sacrifice experimental design^[111]^. In such instances, we would propose that regular and non-invasive collection of condensates may not only enable health monitoring by identification and quantification of important prognostic exhaled biomarkers but also serve to limit and reduce the numbers of animals otherwise necessary for a serial sacrifice approach. Together, our preliminary proteomic analyses substantially support the conclusion that condensates and EBC samples contain exhaled proteins of lung tissue and tumor origin. Future proteomic studies involving larger animal colonies and the collection of larger EBC volumes have the potential to enable unambiguous identification of exhaled tumor proteins or proteins from exhaled tumor EVs.

To date, only a small number of studies have been conducted on mouse exhaled breath that have evaluated the detection of exhaled biomarkers, which may be associated with asthma, non-cystic fibrosis bronchiectasis, and chlorine exposure^[112-114]^. However, to our knowledge, no studies have measured or detected miRNAs in exhaled condensates collected from mice. There have been studies conducted on BAL collected from mouse models of asthma, hyperoxia, and ARDS, which demonstrated that it is rich in miRNAs with potential diagnostic, prognostic, and therapeutic value, and that these miRNAs are contained in EVs originating not only from lung tissue but also from immune and blood cells^[115-117]^. Although a few human studies have suggested that EVs contained in the biofluid lining of the lungs can become aerosolized during normal tidal respiration and be purified from EBC, no studies have evaluated the presence of EVs in mouse EBC^[118-119]^. Thus, using nanoparticle and TEM analyses, we investigated and determined that the EBC of both control and lung tumor-bearing mice contains EVs. Interestingly, whereas a low number of EVs (65-150 nm) could be detected in the EBC collected from control animals (Supplementary Figure 8; *n* = 158 per ml of EBC), we found that this number was ~10-fold greater (Supplementary Figure 8; *n* = 1,450) - and even higher at later stages of disease - for EBC collected from lung tumor-bearing mice. Furthermore, when we used super-resolution nanoimaging, we were able to confirm that the EBC of human lung tumor-bearing mice contained human tumor-derived EVs (positive for CD9, CD63, and CD81). Although we could observe low levels of EVs by TEM in the EBC of control mice, due to the lack of a proper mouse EV validation assay, as per MISEV guidelines, we could not fully validate their identity as being murine cell-derived EVs. Based on our preliminary experiments, we conclude that such validating experiments can be performed but will require very high volumes of EBC (> 10 mL).

Since we determined that the EBC of lung tumor-bearing mice contained human tumor-derived EVs, we sought to evaluate their miRNA cargos and thus customized our EV-CATCHER assay with an anti-human anti-CD63 antibody, which we had previously tested for the purification of human EVs from human plasma and serum^[97]^. We experimentally confirmed the capture and release of intact CD63-GFP^+^ EVs produced in large amounts by our LM-3475 cells *in vitro* prior to purifying low abundance (i.e., compared to tissue culture) human tumor-derived EVs from the EBC of lung tumor-bearing mice. Our miRNA NGS analyses not only support the idea that human lung tumor-derived EV-miRNAs can reliably be detected and quantified in the EBC during tumor progression, but also that the bulk of the miRNA signal originates from exhaled human tumor-derived EVs. We wish to note that although we could non-specifically capture miRNA NGS profiles from the EBC of control mice using our anti-human anti-CD63 EV-CATCHER assay, these profiles only contained 10.5% of the detectable miRNAs with 1,000 times fewer miRNA reads compared to human tumor-derived EVs purified from the EBC of lung tumor-bearing mice. Once we reliably identified human tumor-derived EV-miRNAs consistently detectable at weeks 20, 21, and 22, using qPCR, we demonstrated that a select set of these miRNAs (i.e., miR-222, miR-210, miR-374a, miR-584) could reliably be detected as upregulated within 1-2 weeks post tail vein injection of the metastatic LM-3475 cell line. In contrast, and consistent with studies using other metastatic breast cancer cell lines^[109,110]^, *in vivo* bioluminescent imaging of tumor cells appears significantly detectable 6 weeks after tail vein injection. Although our data suggests that qPCR detection of tumor-specific exhaled EV miRNAs may be more sensitive than bioluminescent imaging of lung tumors in animals (i.e., NGS and qPCR both have lower sensitivity thresholds than standard *in vivo* imaging techniques), further investigation on the sensitivity of this non-invasive detection is required. To date, only one micro-CT scanning mouse study has detected small lung tumors (i.e., as low as 15.4 % of lung volume) by imaging^[120]^. To determine the sensitivity of qPCR detection of early tumors and to determine whether our qPCR detects miRNAs from disseminated cells or from existing small tumor foci, future studies will need to compare micro-CT images of animals at 1-2 weeks post-tail vein injection with the corresponding qPCR signal^[9]^.

Although we experimentally limited our molecular analyses on EBC collected from female mice, considering that breast cancer and secondary lung cancer following primary breast cancer are prevalent in females^[108]^, our findings open a new avenue for the study of lung diseases in animal models, as the non-invasive collection and analysis of their EBC may facilitate the identification and quantification of exhaled miRNAs profiles associated with active, progressing, and/or exacerbated disease. We envision that expanding our approach to study human primary and other secondary lung cancers, in adequately powered animal studies, has the potential to identify relevant exhaled human EV biomarkers^[121-125]^. However, instead of using clonal cell lines, we propose that using patient-derived tumor cells in immuno-competent mice may help investigate the potential diagnostic and/or prognostic value of human-tumor exhaled EV miRNAs and other RNAs^[126]^. Furthermore, since EV-CATCHER can easily be customized to target surface markers of specific EV subpopulations, we foresee that using it to separate lung tumor cell-derived exhaled EVs from immune and innate cell-derived EVs may help further improve the selection of exhaled tumor EVs for the fine-tuned detection of different types of lung cancers. It is important to note that diet, inflammation, and other confounding factors associated with the environment may also be studied in lung cancer animal models to determine whether their effects on tumor growth may be non-invasively detectable in exhaled tumor EVs or exhaled EVs of different cellular origin (i.e., immune cells, lung cells).

Overall, our findings are highly clinically relevant, as the lung represents a central organ for the circulation of blood and is an opportunistic site for metastatic colonization of circulating tumor cells (CTCs), which can originate from several primary tumor organ sites that include breast, colorectal, head-and-neck, urogenital, gynecological and lymphatic cancers^[127-132]^. Considering that these biologically different cell types have the potential to colonize the lung, the analysis of EBC from lung tumor-bearing animal models established using metastatic cancer cells originating from different tissue types may help identify common metastatic miRNAs (e.g., the miR-200 metastatic cluster was detected in both our mouse EBC datasets) as well as primary tumor site-specific miRNAs, which may help with the development of molecular assays for early and non-invasive detection of metastatic lung disease. However, to determine its applicability to human EBC studies, careful evaluation of the type of EBC collection system, proper representation of lung cancer types and stages for adequate power analyses, and most importantly, evaluation of the reproducibility and sensitivity of the purification and quantification assays will be necessary to determine the usefulness of EBC and exhaled tumor EV biomarkers for the non-invasive detection of human lung cancers.

### Limitations of the study

Although our study is novel and provides insights into the collection and analysis of exhaled tumor EVs from tumor-bearing animal models, we have identified several limitations that should be carefully considered when designing future studies. Particularly, we showed that our v2.0 system allowed for the collection of a low number of EVs from control animals, which limited subsequent analyses and molecular validations. Even though we had confirmed the specificity of our anti-human anti-CD63 EV-CATCHER assay, we determined that we could still capture non-specific miRNA signal, likely due to mouse anti-CD63 recognition. This indicates that to conduct species-specific EV purifications, additional anti-human anti-CD63 antibodies will have to be tested to guarantee sole purification of human tumor EVs from mouse EBC. Alternatively, we propose that a targeted anti-GFP EV-CATCHER assay, although not biologically relevant to EVs (i.e., not targeting common EV tetraspanins), could be used in future studies to increase specific selection of exhaled human tumor-derived EVs produced by CD63-GFP^+^ LM-3475 cells, along with GFP fluorescent signal detection to further confirm the identity and origin of CD63-GFP^+^ tumor EVs.

In sum, this study is the first of its kind and it unambiguously demonstrates that exhaled EVs and their miRNA cargos can be purified and quantified from the EBC of lung tumor-bearing animal models to detect aggressive secondary lung cancer of primary breast origin non-invasively.

## DECLARATIONS

### Availability of data and materials

Data supporting the published data will be shared on reasonable requests.

### Copyright

© The Author(s) 2024.
