# Supplementary material for: Non-invasive detection of orthotopic human lung tumors by microRNA expression profiling of mouse exhaled breath condensates and exhaled extracellular vesicles
Source: Extracell Vesicles Circ Nucl Acids. Author manuscript; Available in PMC 2024 Jun 11. (PMC11165456; doi:10.20517/evcna.2023.77)
Supplement: Supplementary [file NIHMS1984606-supplement-Supplementary.pdf]

Supplementary Materials

Non-invasive detection of orthotopic human lung tumors by microRNA expression profiling of mouse exhaled breath condensates and exhaled extracellular vesicles

Megan I. Mitchell[1,6], Iddo Ben-Dov[2], Christina Liu[1], Tao Wang[3], Rachel B. Hazan[4], Thomas L. Bauer[5], Johannes Zakrzewski[1,6], Kathryn Donnelly[1], Kar Chow[6], Junfeng Ma[7], Olivier Loudig[1,6]

[1]Center for Discovery and Innovation, Hackensack Meridian Health, Nutley, NJ 07110, USA.

[2]Laboratory of Medical Transcriptomics, Hadassah-Hebrew University Medical Center, Jerusalem 91120, Israel.

[3]Department of Epidemiology and Population Health, The Albert Einstein College of Medicine, Montefiore Medical Center, Bronx, NY 10461, USA.

[4]Department of Pathology, The Albert Einstein College of Medicine, Montefiore Medical Center, Bronx, NY 10461, USA.

[5]Jersey Shore University Medical Center, Hackensack Meridian Health, Neptune City, NJ 07753, USA.

[6]Hackensack University Medical Center, Hackensack Meridian Health, Hackensack, NJ 07601, USA.

[7]Department of Oncology, Lombardi Comprehensive Cancer Center, Georgetown University Medical Center, Washington, DC 20007, USA.

Correspondence to: Dr. Olivier Loudig, Center for Discovery and Innovation. Hackensack Meridian Health, 111 Ideation Way, Nutley, NJ 07110, USA. E-mail: olivier.loudig@hmh-cdi.org

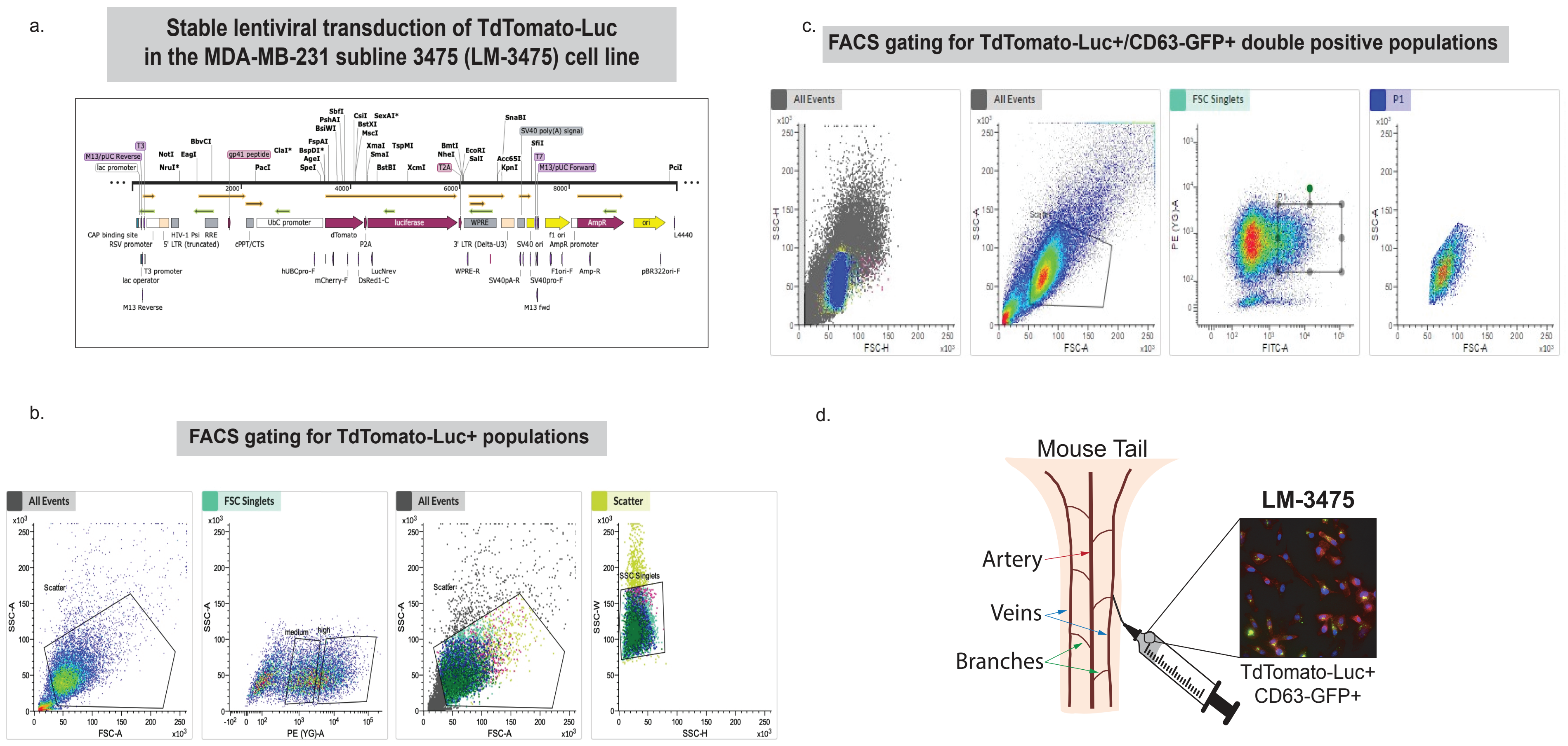

Supplemental Figure 1. Production of double positive MDA-MB-231 subline 3475 cells for TdTomato-Luc and CD63-GFP fluorescent proteins. **a.** Map of the pUC-TdTomato-Luc construct that was stably transduced in LM-3475 cells. **b**. Fluorescence-activated cell sorting (FACS) gating strategy for the clonal selection of high intensity TdTomato-Luc positive cells. **c**. Fluorescence-activated cell sorting (FACS) gating strategy used for the selection of double positive TdTomato-Luc and CD63-GFP LM-3475 cells. As displayed in the bottom right panels we selected double positive cells by gating around the PE (i.e., TdTomato) vs FITC (GFP) population and then selecting the highest intensity cells within this population for sorting (i.e., P1) based on forward and side scatter. **d.** Schematic description of the tail-vein injection process utilized to inoculate individual mice with $1x10^6$ TdTomato-Luc+/CD63-GFP+ MDA-MB-231 subline 3475 cells for the development of lung tumors in athymic BALB/c mice.

a.

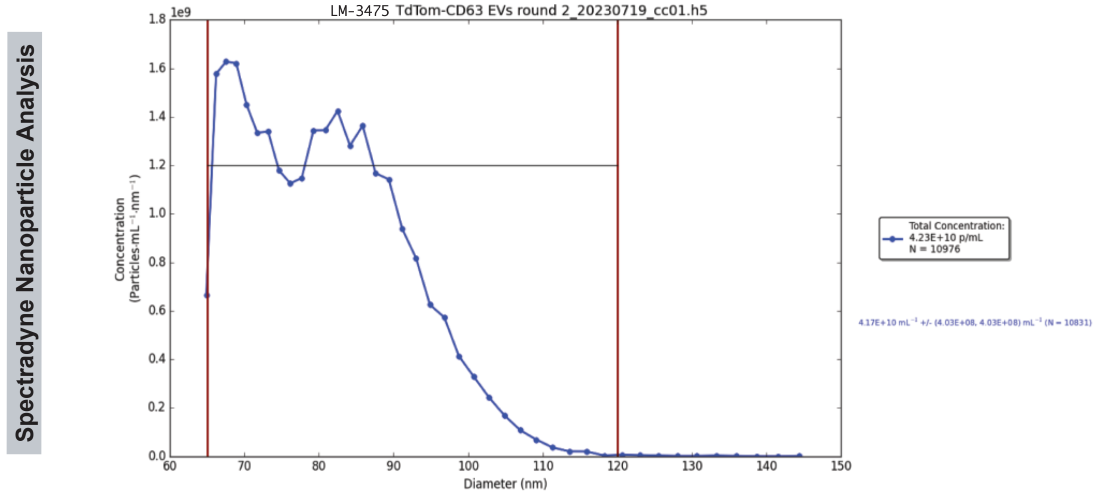

b.

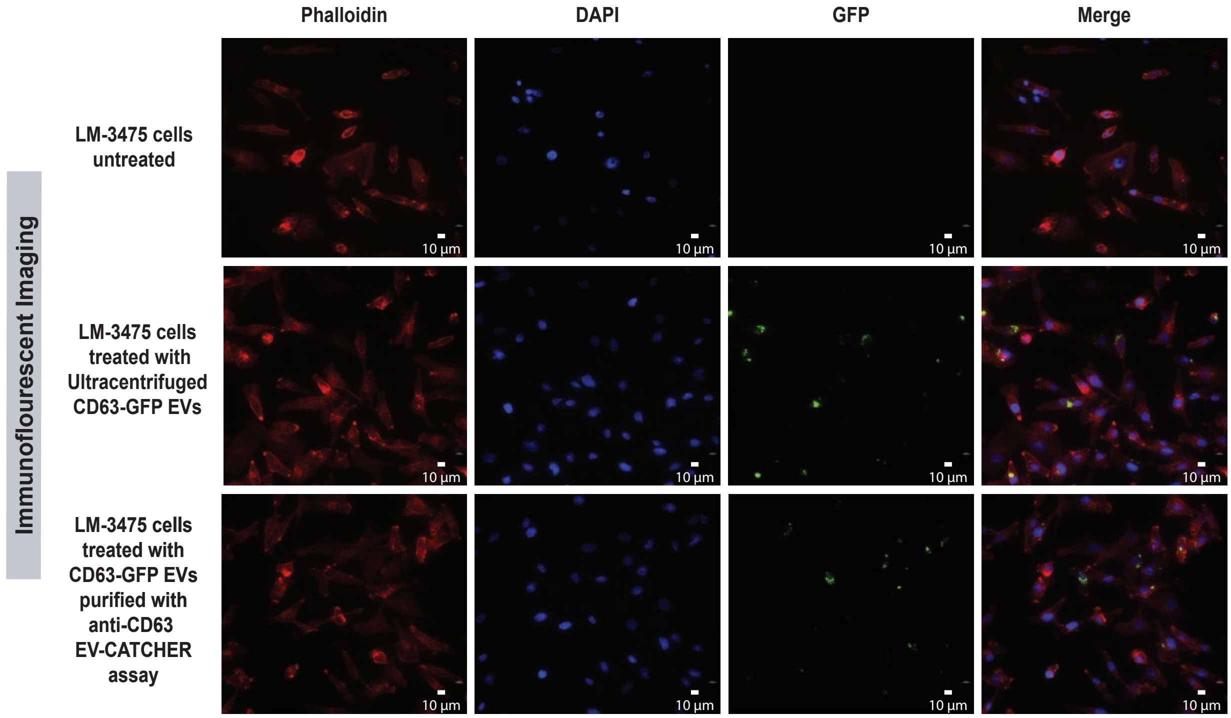

Supplemental Figure 2. Quantification and monitoring the uptake of CD63-GFP EVs produced by LM-3475 cells and purified by ultracentrifugation and the anti-human anti-CD63 EV-CATCHER assay. **a.** Spectradyne nCS1 nanoparticle analysis of LM-3475 derived EVs. The Blue plot in the data plot displays a total of 158 nanoparticles detected in 1 ml of ultracentrifuged from EBC that was pooled from 3 control female mice. The Green plot in the data plot displays a total of 1,450 nanoparticles detected in 1 ml of ultracentrifuged EBC pooled from 3 lung tumor-bearing female mice, collected between weeks 14 and 18. **b.** confocal microscopy of EV uptake in non-transduced LM-3475 cells. ***The upper panels*** display parental non-transduced untreated LM-3475 cells upon phalloidin and DAPI staining, and GFP fluorescent detection, with a merged image of all three. ***The center panels*** display parental non-transduced LM-3475 cells treated for 24 hours with 10^{10} GFP positive EVs produced by LM-3475 cells stably transduced with the pCT-CD63-GFP construct, which were purified by ultracentrifugation, as described in Mitchell M. *et al.* 2021. The phalloidin, DAPI, and GFP fluorescent detection are displayed individually and merged in a single image. Fluorescent detection GFP protein was detected inside LM-3475 cells and indicated uptake of GFP^+-EVs by parental non-transduced LM-3475 cells. ***The lower panels*** display parental non-transduced LM-3475 cells treated for 24 hours with 10^{10} GFP^+-EVs produced by LM-3475 cells stably transduced with the pCT-CD63-GFP construct, which were purified with the anti-human anti-CD63 EV-CATCHER assay previously described in Mitchell M *et al.*, 2021 [97].

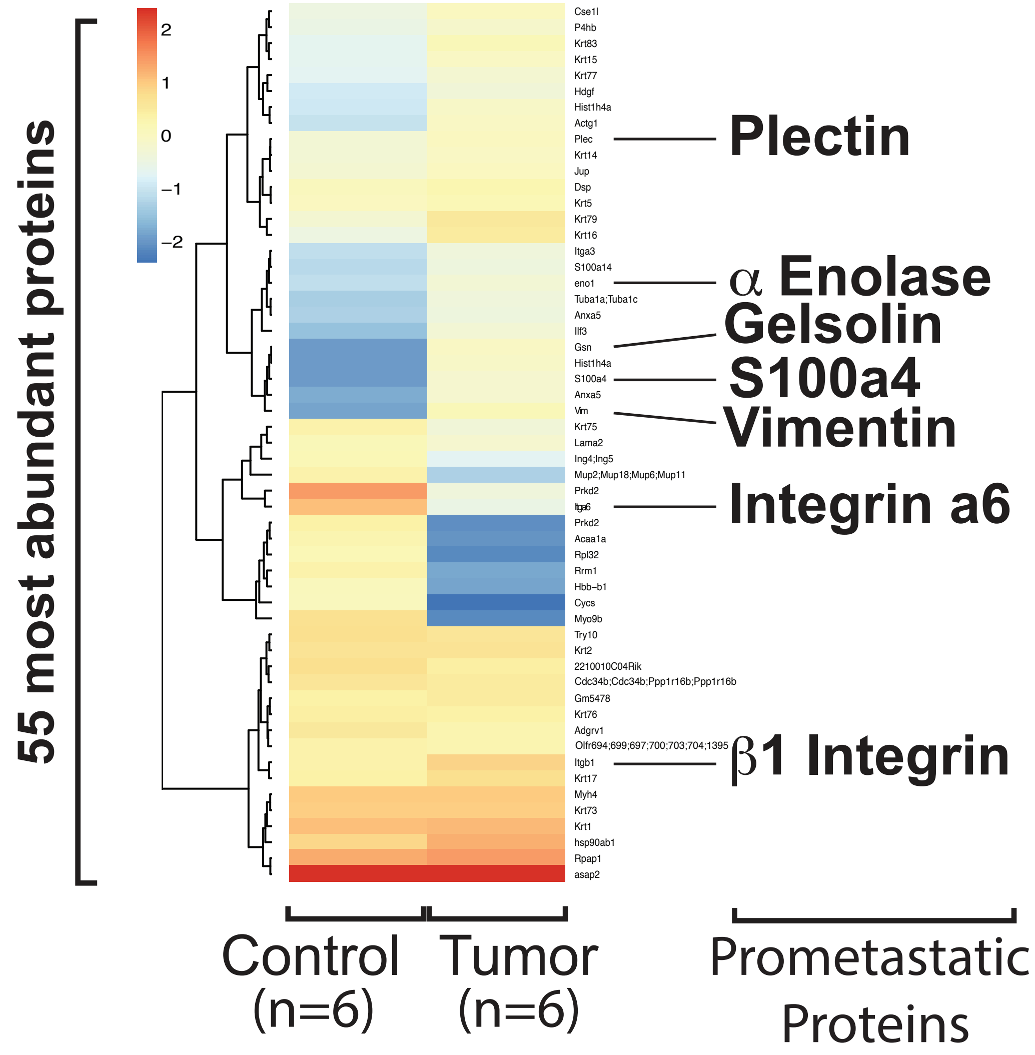

Supplemental Figure 3. Proteomic analysis of EBC from healthy controls and lung tumor-bearing mice. Heatmap analysis of the top 55 most differentially expressed proteins detected using coupling suspension trapping based sample preparation with label-free data-independent acquisition mass spectrometry (S-Trap coupled DIA-MS) [100]. Seven of the top 55 selected proteins have been associated with metastasis in our previous study and are individually enhanced [100].

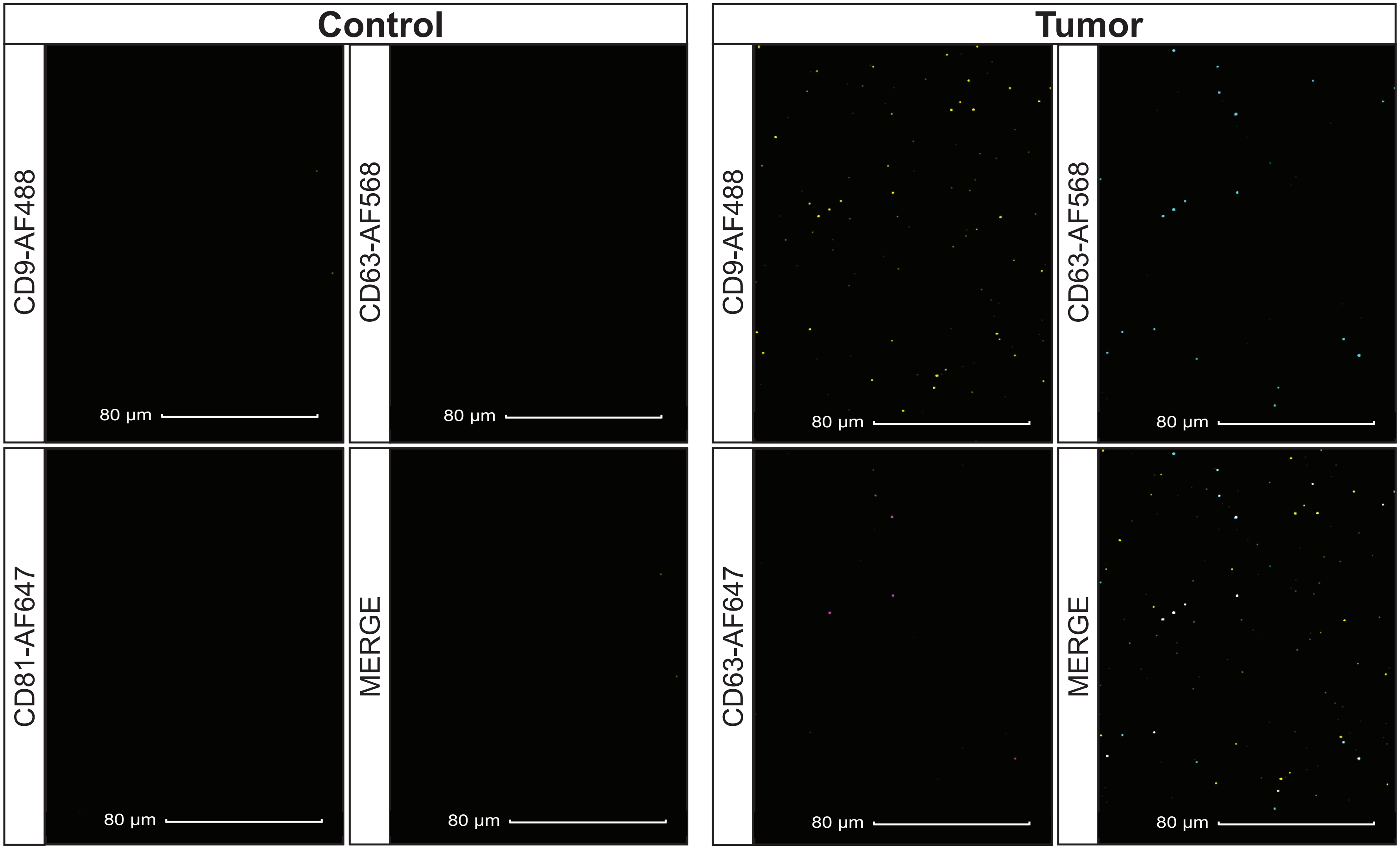

Supplemental Figure 4. Whole field view of the EVs bound to the human EV profiler platform. The ***upper*** images display the entire empty binding field obtained after scanning at the different wavelengths for detection of anti-human anti-CD63 (Green 640 nm), anti-CD9 (yellow 720 nm), and -CD81 (red, 620nm) fluorescent antibodies. The ***lower*** images display the entire field obtained after scanning at the three different wavelengths for detection of anti-human anti-CD63 (Green 640 nm), anti-CD9 (yellow 720 nm), and -CD81 (red, 620nm) fluorescent antibodies. The white dots display EVs triple positive for all three tetraspanins and other single or double colors display EVs positive for one or two tetraspanins.

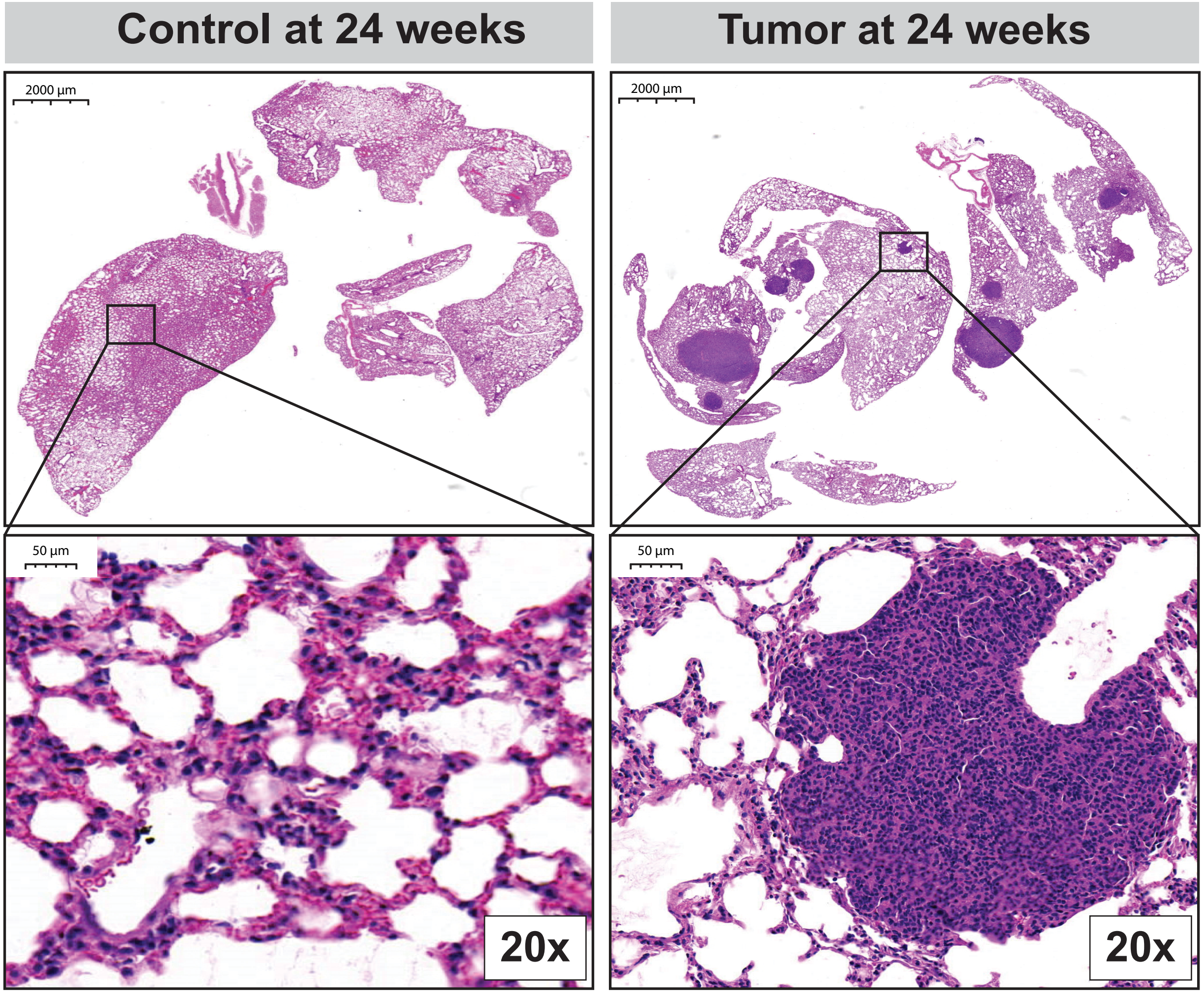

Supplemental Figure 5. Hematoxylin and Eosin (H&E) stained lung tissue section (5µm) from the lung of a control mouse (left panel) displaying normal tissue, and H&E-stained lung tissue section of the of a lung tumor-bearing female mouse (right panel) after 24 weeks. The H&E of the lung tumor bearing mouse (right panel) is showing extensive infiltration of the multi-foci metastatic carcinoma lesions.

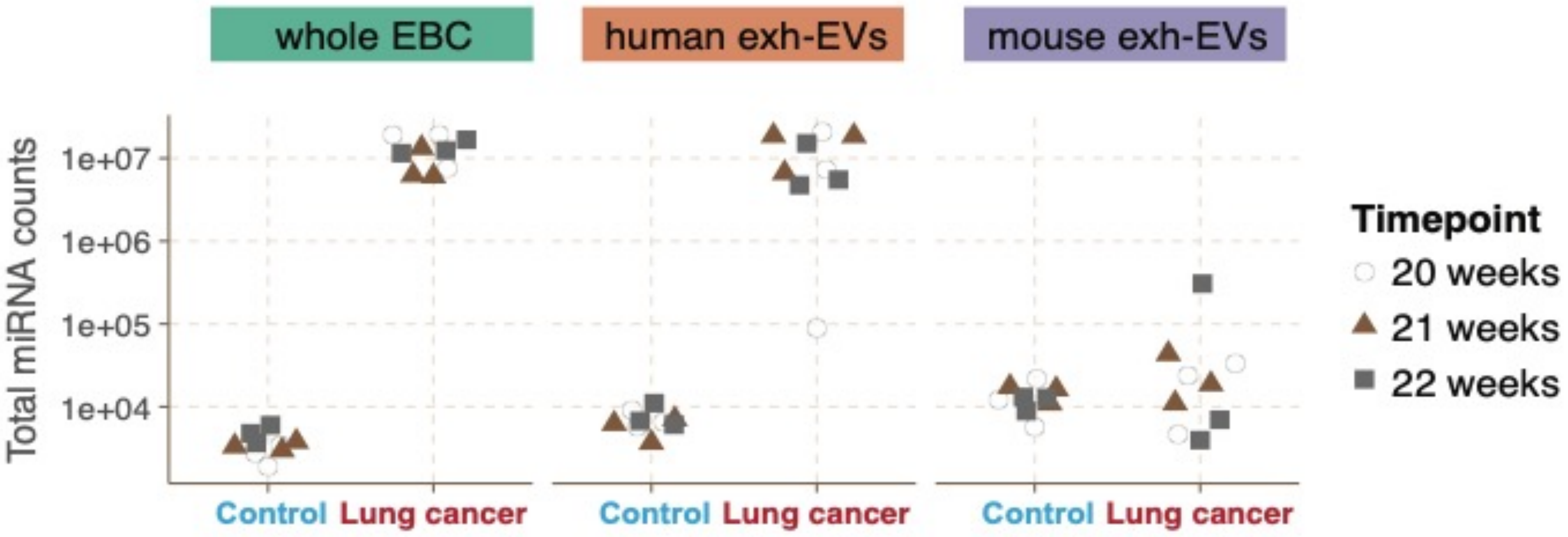

Supplemental Figure 6. Total miRNA read counts for Next Generation Sequencing data from EBC samples. The left graph displays the total miRNA read counts for miRNA NGS libraries obtained from small-RNAs extracted from whole EBC samples (green title) of both control (triplicate measures from 9 mice (3x3)) and lung tumor-bearing animals (triplicate measures from 9 mice (3x3)) collected at weeks 20 (white circle), 21 (brown triangle) and 22 (grey square) on a scale between 104 to 107 total miRNA read counts. The center graph displays the total miRNA read counts for miRNA NGS libraries obtained from small-RNAs extracted from human exhaled EVs purified from EBC samples (orange title) of both control (triplicate measures from 9 mice (3x3)) and lung tumor-bearing animals (triplicate measures from 9 mice (3x3)) collected at weeks 20 (white circle), 21 (brown triangle) and 22 (grey square) on a scale between 104 to 107 total miRNA read counts. The right graph displays the total miRNA read counts for miRNA NGS libraries obtained from small-RNAs extracted from mouse exhaled EVs purified from EBC samples (purple title) of both control (triplicate measures from 9 mice (3x3)) and lung tumor-bearing animals (triplicate measures from 9 mice (3x3)) collected at weeks 20 (white circle), 21 (brown triangle) and 22 (grey square) on a scale between 104 to 107 total miRNA read counts.

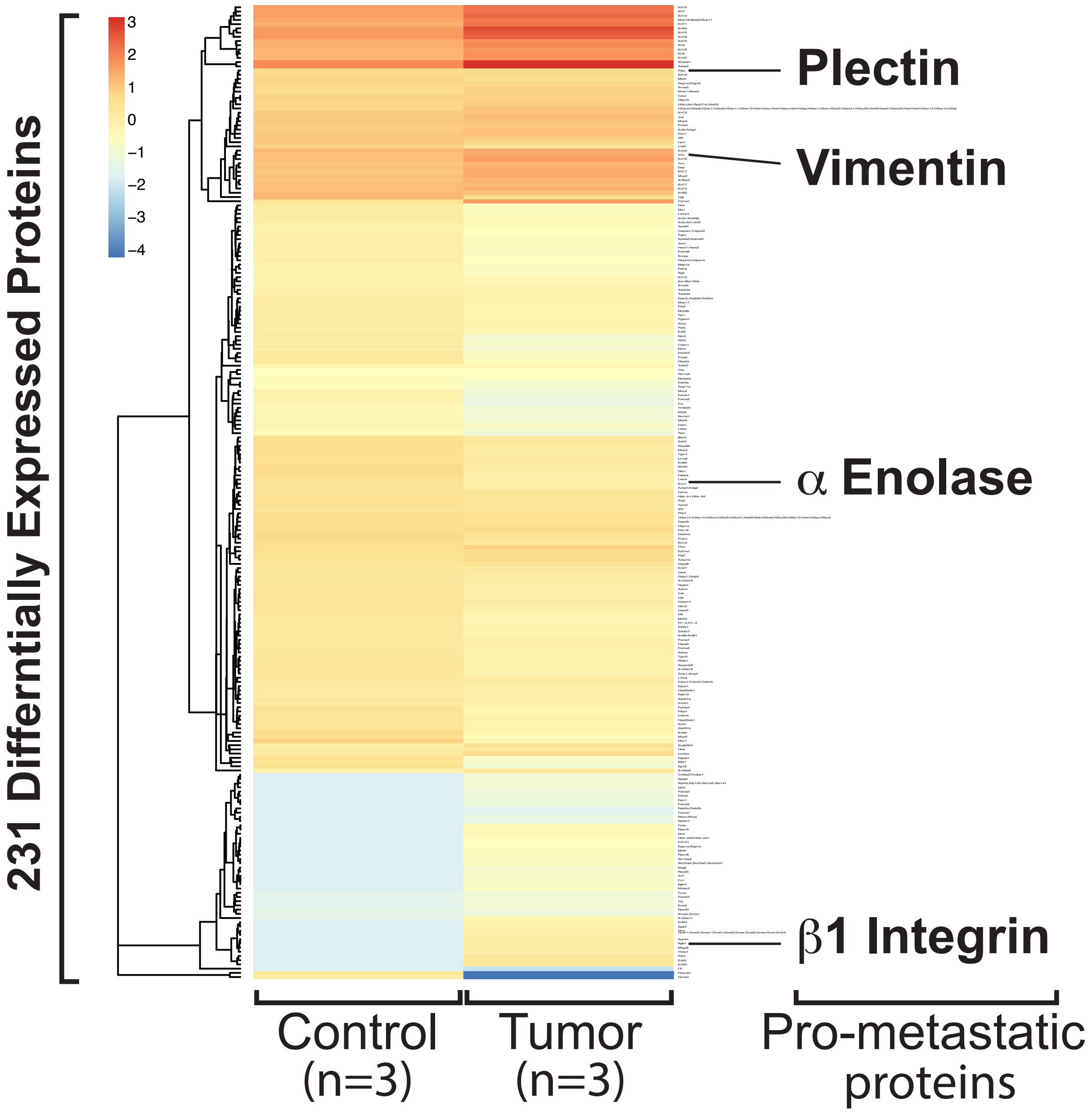

Supplemental Figure 7. Heatmap analysis of all 231 proteins detectable in EBC separately pooled from 3 control and 3 lung tumor-bearing mice, collected at week 10, using coupling suspension trapping based sample preparation with label-free data-independent acquisition mass spectrometry (S-Trap coupled DIA-MS; [100]). Based on all identified proteins, 4 were identified to have known associations with the metastatic process and are individually enhanced, as identified in Fig. 4 and in our previous study [100].

LM-3475 (MDA-MB-231 subline 3475)

Spectradyne Nanoparticle Analysis

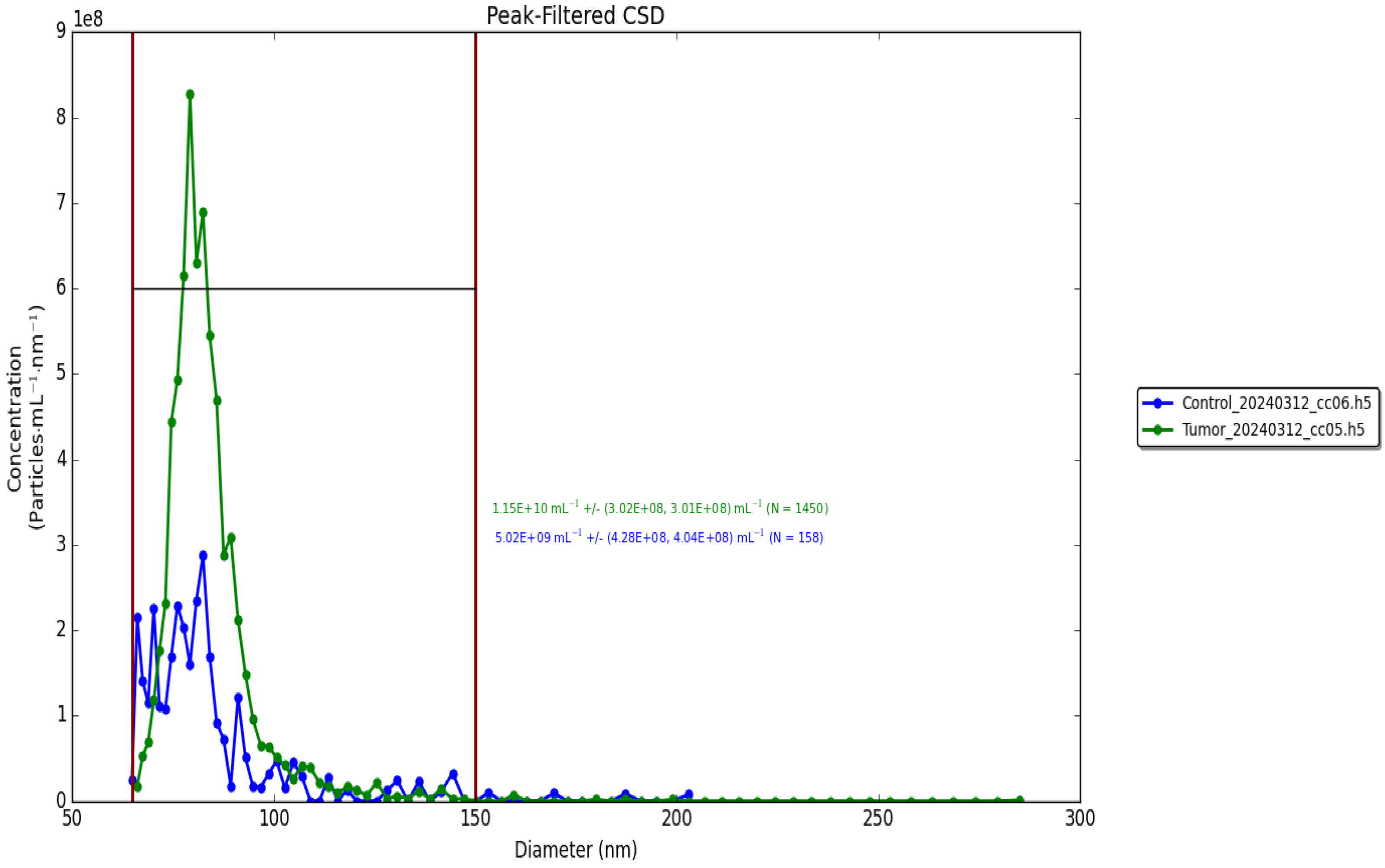

Supplemental Figure 8. Spectradyne nCS1 nanoparticle analysis of EBC. The ***Blue plot*** in the data plot displays a total of 158 nanoparticles detected in 1 ml of ultracentrifuged from EBC that was pooled from 3 control female mice. The ***Green plot*** in the data plot displays a total of 1,450 nanoparticles detected in 1 ml of ultracentrifuged EBC pooled from 3 lung tumor-bearing female mice, collected between weeks 14 and 18.
